# Combining OSMAC Approach and Untargeted Metabolomics for the Identification of New Glycolipids with Potent Antiviral Activity Produced by a Marine *Rhodococcus*

**DOI:** 10.3390/ijms22169055

**Published:** 2021-08-22

**Authors:** Fortunato Palma Esposito, Rosa Giugliano, Gerardo Della Sala, Giovanni Andrea Vitale, Carmine Buonocore, Janardhan Ausuri, Christian Galasso, Daniela Coppola, Gianluigi Franci, Massimiliano Galdiero, Donatella de Pascale

**Affiliations:** 1 Department of Marine Biotechnology Stazione Zoologica Anton Dohrn, Villa Comunale, 80121 Naples, Italy; fortunato.palmaesposito@szn.it (F.P.E.); gerardo.dellasala@szn.it (G.D.S.); giovanniandrea.vitale@szn.it (G.A.V.); carmine.buonocore@szn.it (C.B.); christian.galasso@szn.it (C.G.); daniela.coppola@szn.it (D.C.); 2 Department of Experimental Medicine, University of Campania “Luigi Vanvitelli”, 80138 Naples, Italy; rosa.giugliano@unicampania.it (R.G.); massimiliano.galdiero@unicampania.it (M.G.); 3 Institute of Biochemistry and Cell Biology, National Research Council, 80131 Naples, Italy; janardhan.ausuri@ibbc.cnr.it; 4 Department of Medicine, Surgery and Dentistry “Scuola Medica Salernitana”, University of Salerno, 84081 Baronissi, Italy; gfranci@unisa.it

**Keywords:** trehalolipids, glycolipids, antiviral, antiproliferative, biosurfactant, marine bacteria, OSMAC approach, microbial natural products, mass spectrometry, metabolomics

## Abstract

Natural products of microbial origin have inspired most of the commercial pharmaceuticals, especially those from Actinobacteria. However, the redundancy of molecules in the discovery process represents a serious issue. The untargeted approach, One Strain Many Compounds (OSMAC), is one of the most promising strategies to induce the expression of silent genes, especially when combined with genome mining and advanced metabolomics analysis. In this work, the whole genome of the marine isolate *Rhodococcus* sp. I2R was sequenced and analyzed by antiSMASH for the identification of biosynthetic gene clusters. The strain was cultivated in 22 different growth media and the generated extracts were subjected to metabolomic analysis and functional screening. Notably, only a single growth condition induced the production of unique compounds, which were partially purified and structurally characterized by liquid chromatography high-resolution tandem mass spectrometry (LC-HRMS/MS). This strategy led to identifying a bioactive fraction containing >30 new glycolipids holding unusual functional groups. The active fraction showed a potent antiviral effect against enveloped viruses, such as herpes simplex virus and human coronaviruses, and high antiproliferative activity in PC3 prostate cancer cell line. The identified compounds belong to the biosurfactants class, amphiphilic molecules, which play a crucial role in the biotech and biomedical industry.

## 1. Introduction

Natural products (NPs) of microbial origin, mainly from soil bacteria, have inspired most of our common commercial pharmaceuticals. During the last decade, a shift in focus, from the terrestrial to the marine environment, resulted in the discovery of a wide variety of new industrially relevant metabolites [1]. However, one of the main barriers is represented by the high rediscovery rate of already known metabolites, turning the drug discovery process into a waste of resources [2]. New progresses have been made to overcome these issues, prioritizing the use of talented strains. Recent advances in microbial DNA sequencing technologies allow whole genome sequencing in a rapid and cost-effective way. As a consequence, new tools for genome analysis were developed, such as antiSMASH [3,4], for the identification of biosynthetic gene clusters (BGCs) ranging from polyketide synthase (PKS) and non-ribosomal peptide synthetases (NRPS), to siderophores, post-translationally modified peptides (RiPPs), terpenes, and saccharides. Genome mining approach has revealed that bacterial BGCs far surpass the number of molecules isolated in lab, but often they are silent or cryptic [5,6] and they are not expressed under certain conditions. The concept of “laboratory conditions” plays a fundamental role in the discovery of new NPs. Applying the right stimuli to talented microbes, overexpression of certain metabolites or induction of silent genes will be possible. Among others, the One Strain Many Compounds (OSMAC) approach has been shown to activate BGCs becoming a promising strategy for the development of new bioactive compounds and recent studies highlighted the potential of this approach applied to marine microorganisms [7]. Advancements in the untargeted metabolomics field by using tandem mass spectrometry (MS/MS) and the development of the molecular networking [8] allow a rapid comparison of a high number of metabolites expressed in different conditions. This approach could guide the selection of the unknown compounds produced and facilitate the dereplication step [6]. Selection of promising microbial candidates through genome mining, discovery of the right productive conditions and rapid analysis of metabolic products are factors potentially able to increase the success in the NPs discovery.

Following the state of the art in the discovery of NPs from marine sources, this work aimed to identify novel bioactive compounds, including biosurfactants, from the marine *Rhodococcus* sp. I2R, by using an OSMAC approach to unlock cryptic biosynthetic pathways, coupled with a comprehensive LC-MS/MS (liquid chromatography tandem mass spectrometry)-based untargeted metabolomic analysis and functional screening. Among the other classes of NPs, biosurfactants are well-known amphiphilic molecules, having a wide range of biotechnological applications and including glycolipids, such as the more extensively studied rhamnolipids (RLs), mannosylerythritol lipids (MELs), sophorolipids (SLs), cellobiose lipids (CL), and xylolipids (XL) [9,10]. Chemical industries increasingly recognize the importance of these bio-based compounds as drivers towards a bioeconomy and sustainability [11], as also indicated by the high number of patents regarding the application of biosurfactant molecules [12]. Therefore, there is an emerging interest on marine-derived glycolipids as ingredients for biomedical, biotechnological and personal care formulations [9,13,14,15].

Herein, we report about the fermentative production of a mixture of >30 novel glycolipids from *Rhodococcus* sp. I2R, which were identified as succinic saccharide esters, bearing an uncommon phenylacetate moiety, along with the in vitro investigation of their antiproliferative and antiviral activities.

## 2. Results

### 2.1. Bacterial Isolation and Genome-Based Identification

In the framework of marine biotechnology and bioremediation purposes, marine sediments collected during the EC18 oceanographic campaign in the Southern Tyrrhenian Sea in 2018 and stored at −20 °C were used to isolate marine bacteria on agar plates in presence of 1 mM of phenanthrene. Among others, a rod-shaped orange bacterium named I2R was selected for its capability to grow well on phenanthrene. The preliminary phylogenetic affiliation based on the amplification and analysis of the 16S rRNA region (see Section 4.1) was carried out and identified as *Rhodococcus* sp. As a result, *Rhodococcus* sp. CUA-806 and *Rhodococcus* sp. KRD197 showed the highest similarity (≈99.3%) with *Rhodococcus* sp. I2R. At the species level, a BLAST search against the 16S rRNA gene database, assigned a similarity of 98.59% with *R. yunnanensis*, 98.23% with *R. cerastii*, 98.01% with *R. fascians* and *R. cercidiphylli*. Due to the well-known metabolic potential of this genus, *Rhodococcus* sp. I2R was selected for whole genome sequencing, thereby giving 72 contigs with an overall size of 5.3 Mb and GC content of 64% (Table 1).

The bacterial genome of our strain was compared with those from the closest phylogenetic strains *Rhodococcus* sp. CUA-806 (Genbank MKKD01000002.1) and KRD197 (Genbank JABFAO010000001.1) by average nucleotide identity (ANI) analysis, showing >98% ANI values towards both strains. Therefore, these findings unveil these strains belong to the same species, as the ANI value is higher than the cut-off score (95%) currently accepted for taxonomic inference [16].

### 2.2. Genome Annotation and Biosynthetic Potential Analysis of Rhodococcus sp. I2R

**Figure 1:**
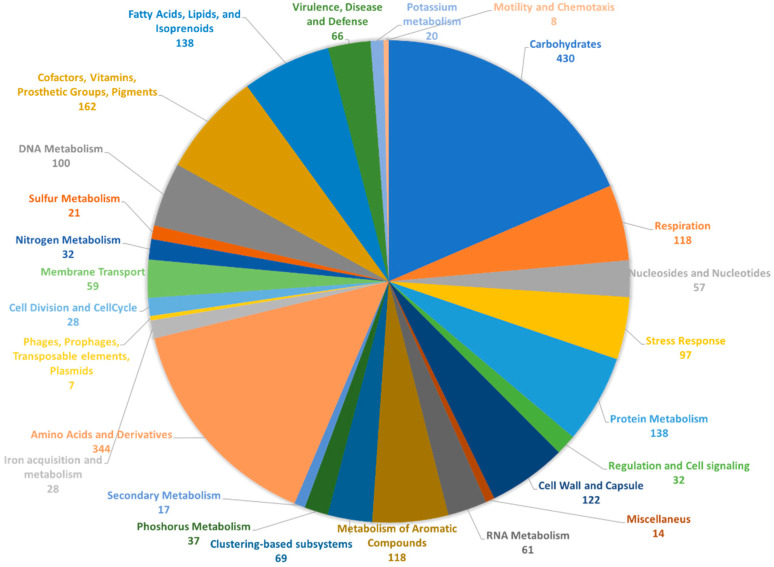
Overview of functional categories assigned to predicted genes from *Rhodococcus* sp. I2R. The whole-genome sequence of the strain I2R was annotated using RAST. The number of genes assigned to each category is indicated.

RAST [17] annotation of the bacterial genome resulted in 5219 coding sequences, of which 2325 (45%) were included in functional categories. An overview of the subsystems for this genome is provided in Figure 1.

The largest functional category is represented by carbohydrates (430 genes), followed by amino acids and derivatives (344 genes), and cofactors, vitamins, prosthetic groups, and pigments (162 genes). Other large categories are represented by fatty acids, lipids, and isoprenoids, and protein metabolism, both with 138 genes. The genes involved in the metabolism of aromatic compounds as well as those for stress response, likely give *Rhodococcus* sp. I2R the ability to grow in polluted or harsh environments [18,19,20]. Indeed, this strain was isolated on phenanthrene agar plates, thus being a good candidate for bioremediation applications.

Aiming to gain a general overview of the biosynthetic potential of *Rhodococcus* sp. I2R, its genome was screened by antiSMASH 6.0 [4], a powerful tool to detect biosynthetic gene clusters (BGCs) involved mainly in the microbial secondary metabolism. As a result, a total of 36 putative BGCs have been identified (Table 2), including pathways encoding the biosynthesis of polyketides, ribosomal and non-ribosomal peptides, terpenes, saccharides, butyrolactones, ectoines, arylpolyenes, and fatty acids. Notably, most of these BGCs displayed partial or no similarity with any known gene clusters, suggesting the possibility of being involved in the production of novel compounds. Interestingly, more than 50% of the detected BGCs (20 out of 36) have been identified as saccharides or saccharides clustered together with NRPS, type-I and type-II PKS, terpene and fatty acid synthases, indicating that this strain is able to produce an extensive repertoire of glycosylated molecules.

The relatively high number of BGCs detected by the antiSMASH analysis of *Rhodococcus* sp. I2R and variability of genes highlighted by genome annotation confirmed the biotechnological interest around this genus and, for this reason, the strain I2R was selected for further studies aimed at the production of novel bioactive metabolites with pharmaceutical and biotechnological applications.

### 2.3. OSMAC-Based Cultivation and Bioactivity Profiling

Twenty-two different growth media were used to cultivate the strain I2R in small-scale cultures (20 mL) following an OSMAC approach in order to elicit the expression of BGCs identified by genome analysis and/or unknown pathways. The growth media selection was guided by a literature survey focused on the cultivation conditions of other reported *Rhodococcus* spp., and more in general, actinobacteria, leading to the production of bioactive metabolites [21,22,23,24]. Culture media were designed to explore the effects of different carbon and nitrogen sources and their concentrations on secondary metabolite biosynthesis, under high and low nutrient conditions. After 6 days of incubation at 20 °C (late stationary phase), *Rhodococcus* sp. I2R was able to grow in all tested media with visible variations in pigmentation. Cultures were treated to obtain crude extracts from the exhausted broths. The yield of extracts ranged from 1 mg to a maximum of 5 mg. The generated extracts were subjected to functional screening, including antiviral, anticancer, and biosurfactant assays. Screening results are described below.

#### 2.3.1. Antiviral Activity

**Figure 2:**
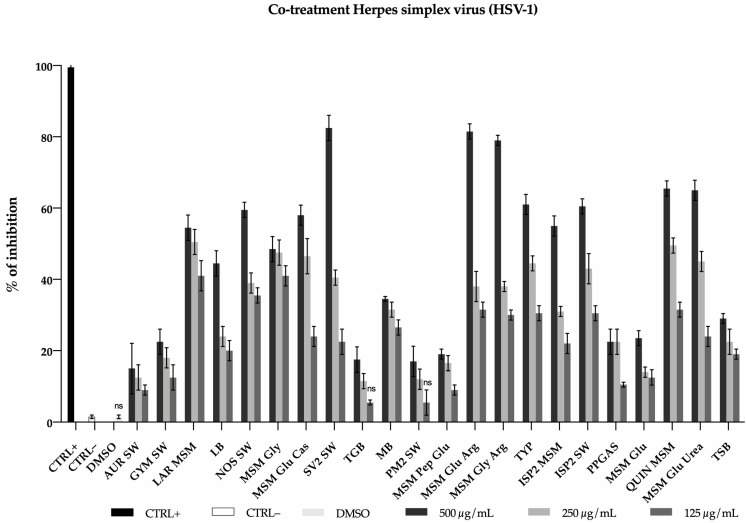
Evaluation of the antiviral activity of 22 crude extracts derived from *Rhodococcus* sp. I2R towards herpes simplex virus type 1 (HSV-1). The percentage of inhibition was calculated by counting the number of plaques obtained in the presence of extract compared to the untreated virus (CTRL–). The Greco extract [25] at 50 µg/mL was used as a positive control (CTRL+). Data are means of three independent experiments. Statistical significances are referred to the negative control (CTRL–). Differences between groups were determined by analysis of variance (ANOVA) and Dunnett’s test was used for multiple comparisons with the control. Statistically not significant values are indicated with “ns”, otherwise *p* < 0.005.

The twenty-two extracts obtained from the OSMAC were tested for the antiviral activity. The Vero cells were treated with the extracts at different concentrations (125, 250, and 500 µg/mL) and simultaneously infected with herpes simplex virus 1 (HSV-1). The percentage of inhibition was calculated by comparing the antiviral effect of the tested extracts with the untreated virus. Out of 22 samples tested, only 3 (SV2 SW, MSM Glu Arg, MSM Gly Arg) showed promising activity against HSV-1, with 80% and 40% inhibition at concentration of 500 and 250 µg/mL, respectively (Figure 2).

#### 2.3.2. Anticancer Activity

**Figure 3:**
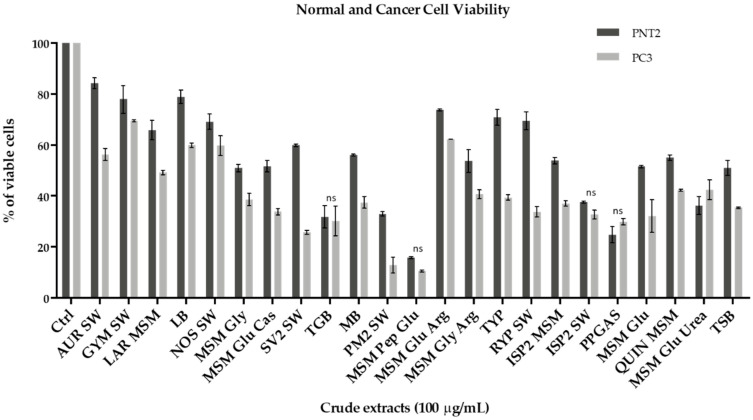
MTT cell viability assay on PNT2 and PC3 cell lines. Histograms represent the percentages of viable cells after 48 h of treatment with 100 µg/mL of crude extracts; assays were performed in triplicate. Cells treated with DMSO vehicle were used as control and correspond to 100% of cell viability. Statistical significances are referred to normal and cancer lines within the same group. Differences between groups were determined by analysis of variance (ANOVA) and Bonferroni test was used as post hoc test. Statistically not significant values are indicated with “ns”, otherwise *p* < 0.005.

The 22 total extracts of *Rhodococcus* sp. I2R were assessed for potential antiproliferative effects on PC3 human prostatic carcinoma cell line and its normal counterpart PNT2 (normal prostatic epithelial cells), by using the MTT assay. PC3 and PNT2 cells were incubated with different doses of crude extracts (1–10–100 µg/mL), however only the highest concentration showed moderate or strong antiproliferative effect (Figure 3 and Figure S1). All the extracts inhibited PC3 cancer cell growth after 48 h, with cell viability ranging from 10 to 69%. In particular, PC3 viability was negatively and significantly affected after exposure to the following samples: SV2 SW (25%), PM2 SW (13%), MSM Pep Glu (10%). Among these three samples, SV2 SW was shown to exert the most selective antiproliferative activity, as unveiling lower cytotoxicity (2.4 times less) towards PNT2 cells (60% of viable cells) (Figure 3 and Figure S2).

#### 2.3.3. Biosurfactant Activity

**Figure 4:**
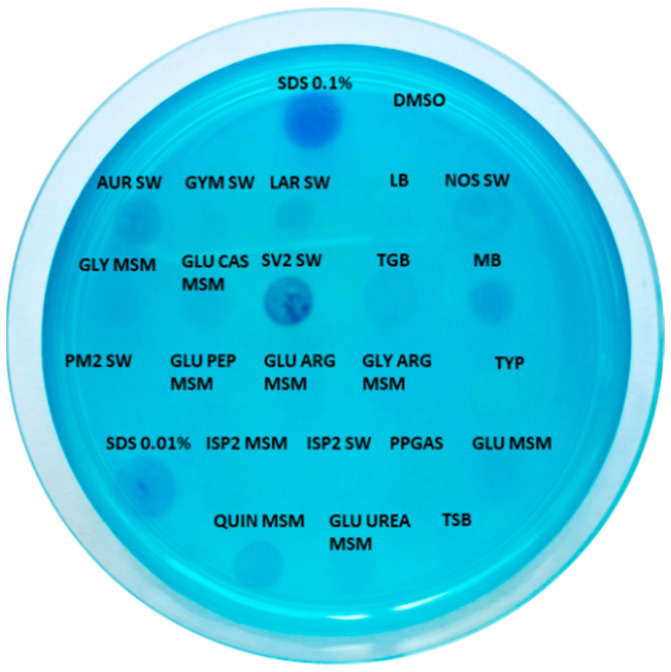
Biosurfactant activity of all extracts (25 mg/mL) on a CTAB agar plate. Blue halos indicate positivity to the test. SDS 0.1% and 0.01% are positive controls, while DMSO vehicle was used as negative control.

The obtained crude extracts were screened for biosurfactant activity using the CTAB (cetyltrimethylammonium bromide) agar method [26] to detect the production of anionic biosurfactants. The presence of such molecules is easily detected by the formation of dark blue halos. Among extracts showing a potential biosurfactant activity (Figure 4), the sample SV2 SW displayed the highest effect, which resulted to be comparable to that of 0.1% SDS and higher than that of 0.01% SDS positive controls. DMSO vehicle was used as negative control.

### 2.4. Molecular Networking Analysis of Rhodococcus sp. I2R Metabolism under Different Culture Conditions

**Figure 5:**
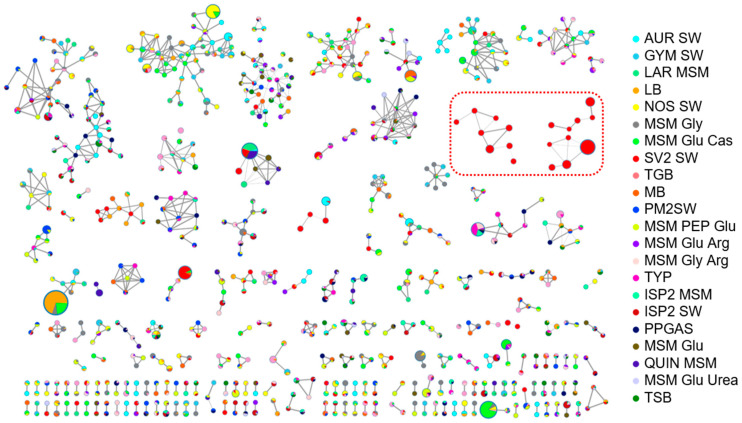
Molecular networking overview of crude extracts produced by *Rhodococcus* sp. I2R in 22 different culture media. Biosynthesis of two molecular clusters (circled in red) was triggered exclusively in the SV2 SW medium. Each color corresponds to a specific growth condition. Node size is relative to the areas of the relevant peaks in the extracted-ion chromatograms from the LC-MS run and edge thickness is relative to the cosine score similarity.

In order to highlight differences in the *Rhodococcus* sp. I2R metabolism caused by different nutrient regimes, the 22 extracts were subjected to low-resolution LC-MS/MS analysis in the negative ion detection mode (Figure S3). Raw MS data were preprocessed by MZmine [27] and submitted to the online platform GNPS [8] to create a molecular network (MN) by using the Feature-based Networking tool (FBMN) [28]. As a result, a wide distribution of related nodes has been detected and the MN derived from the SV2 SW condition revealed the presence of two molecular clusters showing unique nodes (Figure 5), which have been proved later to be glycolipids, and therefore biosurfactant molecules, by liquid chromatography high-resolution tandem mass spectrometry (LC-HRMS/MS) (see Section 2.7). Notably, the SV2 SW organic extract was also shown to exhibit the most interesting bioactivity profile during functional screening (see Section 2.3).

### 2.5. Bioassay-Guided Fractionation of the SV2 SW Crude Extract

Based on the metabolomic and functional screening results, sample SV2 SW was further investigated to identify the active compounds. *Rhodococcus* sp. I2R was scaled-up and cultivated in 400 mL of SV2 SW medium for 6 days, centrifuged and the supernatant extracted by EtOAc. The crude extract (45 mg) was fractionated over a C18 SPE cartridge by using an H_2_O/MeOH gradient to yield four fractions (100% H_2_O, 50% MeOH, 90% MeOH, 100% MeOH). Each eluted fraction, excluding water, was dried and weighted obtaining 6.3 mg of 50% MeOH, 6 mg of 90% MeOH and finally 2 mg of 100% MeOH fraction. Again, the samples were subjected to low-resolution LC-MS/MS and functional screening (using DMSO as vehicle) in order to identify the active fraction and further characterize the metabolites.

#### 2.5.1. Antiviral Activity Validation

**Figure 6:**
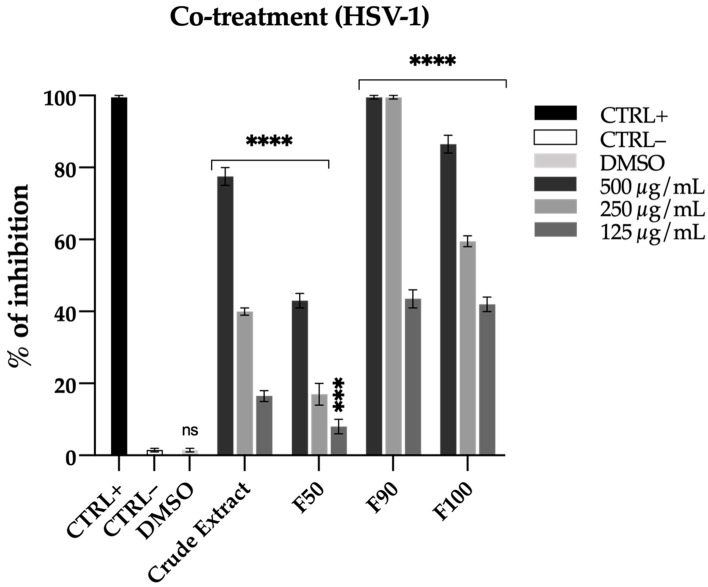
Antiviral effects of SV2 SW fractions against herpes simplex virus 1 (HSV-1) in co-treatment experiments. Vero cells were simultaneously infected with HSV-1 and treated with DMSO vehicle and different concentrations (125, 250, and 500 µg/mL) of the SV2 SW crude extract and fractions F50, F90, and F100. The negative control is represented by untreated cells. The Greco extract at 50 µg/mL was used as positive control (CTRL+). Data are means of three independent experiments. Statistical significances are referred to the negative control (untreated cells). Differences between groups were determined by analysis of variance (ANOVA) and Dunnett’s test was used for multiple comparisons with the control. *** *p* < 0.0002, **** *p* < 0.0001, ns (not significant).

The observed antiviral activity was particularly relevant, as shown by Figure 6. Indeed, in co-treatment experiments where Vero cells were simultaneously infected with HSV-1 and exposed to increasing doses of SV2 SW fractions, the 90% MeOH fraction (F90) unveiled a potent antiviral effect, showing 100% inhibition at 500 and 250 µg/mL concentrations and about 40% inhibition at a concentration of 125 µg/mL. The 100% MeOH fraction (F100) also showed a moderate percentage of inhibition of 80, 60, 40% at concentrations of 500, 250 and 125 µg/mL respectively. Fraction 50% MeOH (F50) did not elicit significant effect. These results clearly indicate that the F90 antiviral activity is always higher than that exerted by the crude extract (and the other fractions), thus suggesting this fraction to be enriched of antiviral compounds (Figure 6).

In the light of these findings, we next pre-treated HSV-1 with the most active fraction F90 and evaluated its infectivity in Vero cells by plaque assay in order to assess whether the loss of viral titer could be ascribed to inhibition of viral replication and/or virus inactivation mechanisms.

**Figure 7:**
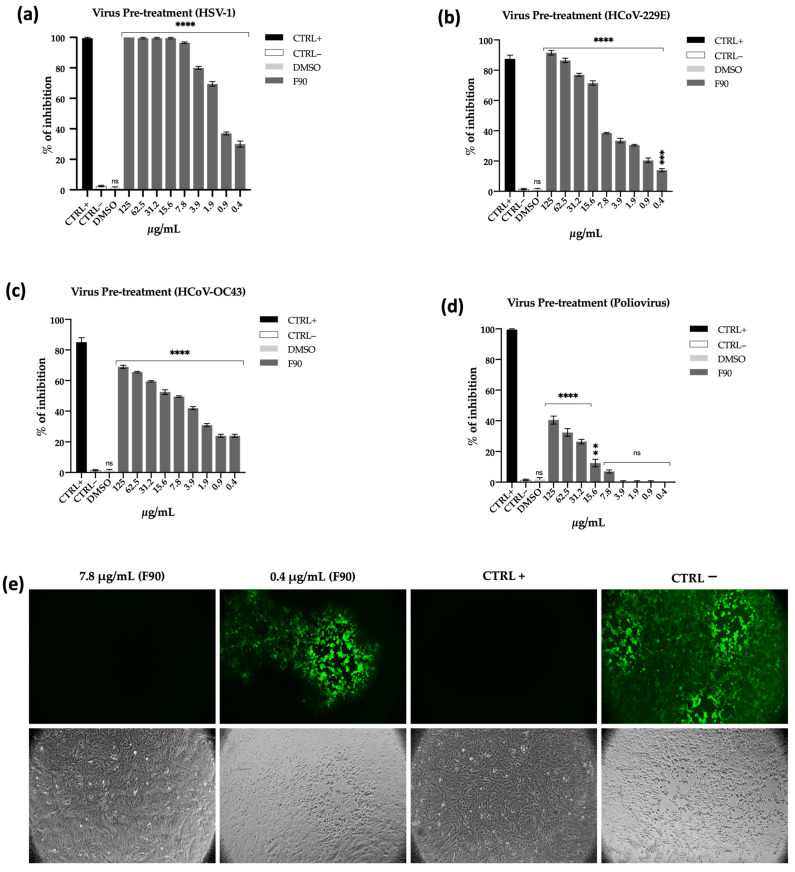
Antiviral activity of fraction 90% MeOH (F90). The antiviral activity of F90 was evaluated against three enveloped viruses: herpes simplex virus type 1 (HSV-1) (**a**), human coronavirus 229E (HCoV-229E) (**b**), human coronavirus OC43 (HCoV-OC43) (**c**), and the envelope-free Poliovirus PV-1 (**d**). F90 was tested, ranging from 125 to 0.4 µg/mL. (**e**) Green Fluorescent Protein herpes simplex virus type 1 (GFP HSV-1) was used to confirm the antiviral activity of F90 at 7.8 µg/mL (absence of fluorescence as CTRL+), while around 30% of inhibition is displayed at 0.4 µg/mL (presence of fluorescence). Greco extract and Pleconaril were used as positive controls (CTRL+). Untreated cells were used as negative control (CTRL–). DMSO was used as an internal negative control. Data are means of three independent experiments. Statistical significances are referred to the negative control. Differences between groups were determined by analysis of variance (ANOVA) and Dunnett’s test was used for multiple comparisons with the control. ** *p* < 0.0021; *** *p* < 0.0002, **** *p* < 0.0001, ns (not significant).

**Figure 8:**
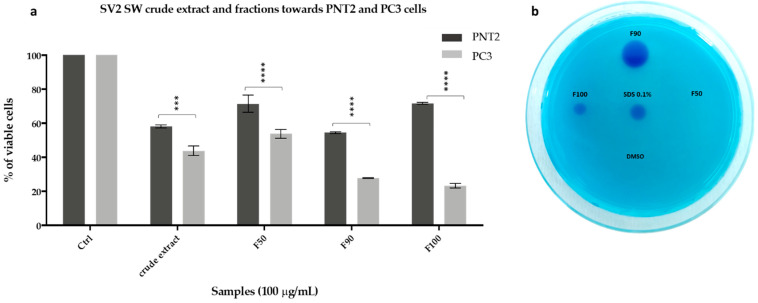
(**a**) MTT cell viability assay on PNT2 and PC3 cell lines. Histograms represent the percentages of viable cells after 48 h of treatment with 100 µg/mL of SV2 SW extract and fractions F50, F90, and F100. Data are means of three independent experiments. Statistical significances are referred to normal and cancer lines within the same group. Differences between groups were determined by analysis of variance (ANOVA) and Bonferroni test was used as post hoc test. *** *p* < 0.0002, **** *p* < 0.0001 (**b**) CTAB agar assay of SV2 SW fractions assessed at 25 mg/mL. SDS at the indicated concentrations (0.1% and 0.01%) was used as positive control, whereas DMSO vehicle as negative control.

For this purpose, the virus was incubated with F90 for 1 h at 37 °C, then the mixture was titrated on monolayer cells. As reported in Figure 7a, preincubation of HSV-1 with F90 completely abolished viral infectivity up to 7.8 µg/mL concentration. However, a significant reduction of infectious virions could be observed even at lower concentrations, thus detecting 80% viral inhibition at 3.9 µg/mL and 70% at 1.8 µg/mL. Taking into account the high surfactant activity shown by F90 in CTAB assay (Figure 8b), these findings suggest that the antiviral action is likely due to a physicochemical interaction of biosurfactant compounds with the virus lipid membrane leading to envelope damage.

Aiming to understand if the F90 fraction was active against HSV-1 in a selective manner, we tested its activity against a broader panel of viruses, including the enveloped human coronaviruses 229E (HCoV-229E) and OC43 (HCoV-OC43), the latter belonging to the genus β-coronavirus and closely related to SARS-CoV-2, and the naked (envelope-free) Poliovirus PV-1 (all viruses used are summarized in Section 4.5.1). In the same way as above, a viral suspension of HCoV-229E and Poliovirus PV-1 was preincubated at 37 °C for 1 h with F90 and then the mixture was used to infect cells. After 24 and 48 h infection for HCoV-229E and sb1, respectively, the plates were washed and the number of plaques counted. On the other hand, to assess in vitro susceptibility of HCoV-OC43 to the F90 fraction, a viral cytopathic effect inhibition assay was performed and cell viability was monitored through the MTT assay. 

Our results demonstrated that also in the case of HCoV-229E the antiviral activity was very high (Figure 7b), with a percentage of inhibition of 95 and 90% at 125 and 62.5 µg/mL. At lower concentrations (31.2 and 15.6 µg/mL) the reported activity was 75 and 70%, reaching 20% of inhibition at 0.4 µg/mL. Positive inhibition effect was also detected against the β-coronavirus HCoV-OC43 (Figure 7c), showing more than 60% inhibition at concentration between 125 and 31.2 µg/mL, gradually decreasing the effect at lower concentrations. As expected for membrane-active biosurfactants, the antiviral effect against the envelope-free Poliovirus PV-1 was considerably reduced, showing 40% and 30% of inhibition at the highest concentrations (Figure 7d). These observations provided further evidence that F90 can inhibit viral infectivity by disrupting membrane integrity or interfering with membrane components.

The antiviral effect of F90 was confirmed by fluorescent microscopy analysis using a Green Fluorescent Protein-modified herpes virus. As clearly showed by Figure 7e, at a concentration of 7.8 µg/mL the active fraction completely inhibited virus entry into cells and this is reflected by the absence of luminescence. On the contrary, lowering the sample concentration (0.4 µg/mL) the virus was able to partially infect the cells as revealed by the detected fluorescence. Inhibition of the cytopathic effect induced by the virus following treatment with the F90 has been evaluated through the MTT assay and did not show a relevant effect (Figure S4).

Taken together, these results demonstrated that the antiviral action of the 90% MeOH fraction was directed to the viral particles presumably through a detergent-like mechanism.

#### 2.5.2. Antiproliferative and Biosurfactant Activity Validation

The SV2 SW fractions were tested to validate the antiproliferative and biosurfactant activity (Figure 8). The results demonstrated that F90 and F100 fractions exerted a strong antiproliferative effect as reducing PC3 cell viability below 30% at 100 µg/mL. PNT2 cells showed higher cell viability after exposure to the same treatments (55 and 72% after incubation with F90 and F100, respectively). Interestingly, lower SV2 SW concentrations (1 and 10 µg/mL) exhibited selective antiproliferative effects in PC3 cancer cells (Figures S5 and S6). Similarly, a biosurfactant effect was elicited by the same fractions using the CTAB agar assay.

### 2.6. Structure Prediction of Novel Succinic Saccharide Esters

**Figure 9:**
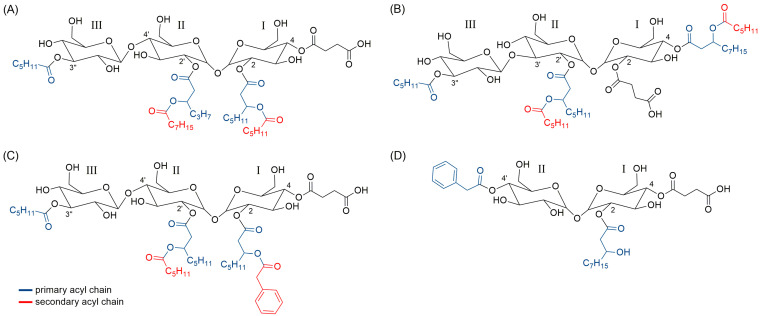
Succinoyl saccharide esters from *Rhodococcus* species. (**A**,**B**) Chemical structures of the succinoyl trisaccharide tetraesters isolated from the deep-sea *Rhodococcus* sp. BS-15 (**A**) and an unclassified *Rhodococcus* species, annotated as ‘isolate Q’ (**B**). These biosurfactants have a hydrophilic backbone consisting of two α,α − 1,1 glycosidic linked glucose units, i.e., trehalose, which bears a third β-glucose unit (III) with a hexanoate at C3″. The trehalose moiety is acylated at C2/C2′ and C4/C4′ with succinate and *O*-ester-linked acyloxyacyl motifs. (**C**,**D**) Putative structures of a representative succinoyl trisaccharide tetraester (**C**) and a succinoyl disaccharide triester (**D**) from *Rhodococcus* sp. I2R discussed in this study. Positions of substituents linked to the saccharide backbone have been assigned according to compound **A**.

For structural prediction of congeners included in the two unique biosurfactant clusters detected by MN (Figure 5), fraction F90 was subjected to LC-HRMS/MS on an LTQ Orbitrap instrument. Particularly, a survey full MS scan (performed both in positive and negative ion detection modes) unveiled a complex mixture of closely related compounds, including several series of molecules differing in degree of unsaturation and oxidation and/or by methylene units. Most intense ions (Table 3) were selected for fragmentation for structural prediction in the negative ion detection mode. The high-resolution ESI-MS of deprotonated [M − H]^–^ and protonated [M + H]^+^ molecular ions were in accordance with molecular formulas reported in Table 3 and Table S1 (mass accuracy ≤5 ppm and ≤1 ppm in negative and positive ion detection modes, respectively). An in-depth analysis of the HRMS/MS fragmentation patterns of the selected molecular ions suggested the F90 to be mainly composed of di- and tri-saccharide succinic esters, as sharing almost the same fragmentation pathways with succinoyl trehalolipids from an unclassified *Rhodococcus* (annotated as “isolate Q”) [29] and the deep-sea *Rhodococcus* sp. BS-15 [30]. Based upon these observations, we hypothesized our compounds to be succinoyl trehalolipids, considering that (a) *Rhodococcus* species are widely reported to produce this class of biosurfactants [31] and (b) our isolate possesses the biosynthetic machinery for trehalose (see Section 2.7). The structure of the glucotriose lipid biosurfactant determined by Konishi et al. [30] was used as a model to tentatively assign positions of substituents linked directly to the saccharide backbone of glycolipids discussed in this study (Figure 9).

**Figure 10:**
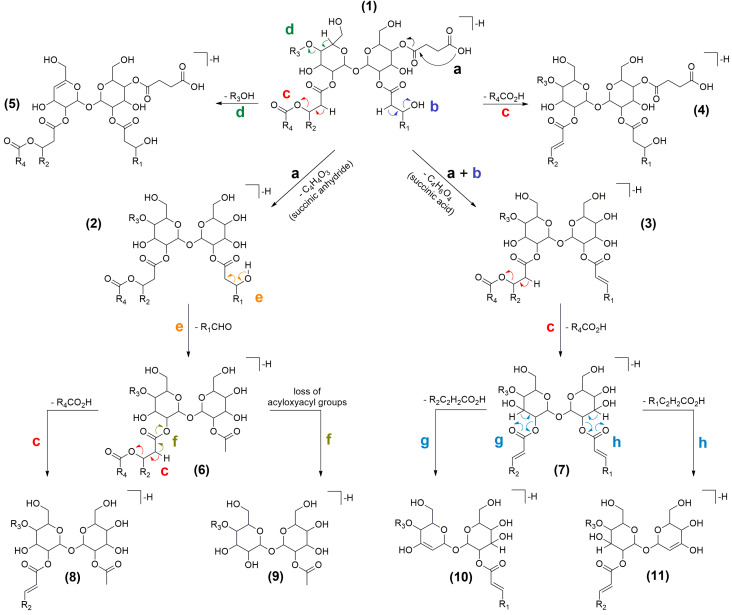
Proposed ESI-fragmentation pathways of succinic saccharide esters discussed in this study.

MS/MS spectra of putative succinoyl trehalolipids unveiled the presence of the fragment ion at *m/z* 341.1084 (C_12_H_21_O_11_^−^), together with ions deriving from neutral losses of one and two water molecules at *m/z* 323.0978 and 305.0873, respectively, which were consistent with a disaccharide unit consisting of two hexose rings (presumably a trehalose unit). Similarly, compounds displaying fragments at *m/z* 503.1612 (C_18_H_31_O_16_^−^), 485.1506 (−H_2_O), and 467.1401 (−2 H_2_O) were predicted to bear a trisaccharide unit made up of three hexose moieties (most probably trehalose linked to a third glucose unit). Moreover, in all reported compounds, the saccharide backbone contained an ester-linked succinate, as shown by fragmentation reactions resulting in losses of succinic anhydride (C_4_H_4_O_3_, 100.0160 amu) and/or succinic acid (C_4_H_6_O_4_, 118.0266 amu) (Figure 10, fragmentations *a* and *a + b*).

A careful examination of the MS tandem spectra allowed to establish the acylation patterns of the di- and tri-saccharide succinic esters as described in detail in Table 3, as ESI fragmentation mostly generated ions as a result of side-chain losses rather than by cleavage of the carbohydrate skeleton. Succinoyl glycolipids were grouped in di-, tri-, and tetraesters according to the number of primary acyl chains, i.e., acyl groups directly linked to the sugar moiety (Figure 9). Most of the detected di- and tri-saccharide succinic esters were found to be acylated with hydroxylated medium-chain fatty acids (OH-FAs), which bore either secondary acyl chains through *O*-ester linkage (Figure 9) or free hydroxy groups.

The presence of medium-chain OH-FAs was suggested by fragment ions deriving from the elimination of (a) the secondary acyl group (fragmentation *c*, Figure 10) or (b) a water molecule (fragmentation *b*, Figure 10), following hydrogen removal assisted by heteroatoms with lone electron pairs or negatively charged groups, acting as Lewis base [32]. Moreover, these OH-FAs were supposed to be hydroxylated at the β-position, as those bearing a free OH group gave diagnostic fragments generated by a typical McLafferty-type rearrangement leading to the cleavage of the Cα-Cβ bond (fragmentation *e*, Figure 10) [33]. Overall, anions **10** and **11** (Figure 10) allowed us to determine the chain length of the OH-FAs directly linked to the sugar unit (fragmentation *g* and *h*, Figure 10).

To confirm identity of 3-OH-FAs, fatty acid methyl esters (FAMEs) were prepared from the 90% MeOH fraction by methanolysis with H_2_SO_4_ and analyzed by GC-MS (Table 4). GC-MS analysis showed OH-FAMEs to have the base ion at *m/e* = 103, characteristic for 3-hydroxylated FAMEs [34]. As molecular ions of OH-FAMEs are usually too weak to be distinguished from background noise, chain lengths were indirectly established by mass fragments at *m/e* = M^+^ − 50, due to the loss of water and methanol [34]. Notably, β-hydroxyoctanoate and β-hydroxydecanoate were the major compounds, as already reported by Esch et al. [29]; in addition, branched OH-FAs were also detected, even if in much lower amount as compared to their straight-chain counterparts (Table 4). Branched isomers were tentatively identified based on their retention times (and “busier” spectra) with respect to those of the analogous straight-chain esters [35]. Interestingly, an uncommon 3-OH FAME corresponding to methyl 3-OH-4-phenylbutanoate was identified using the NIST11 mass spectral library (Figure S7) [36], in full accordance with ESI MS/MS data showing succinic saccharide esters featuring this unusual acyl chain (Figure S8).

Succinic saccharide esters reported in Table 1 were found to bear hexanoate and phenylacetate units, as shown, respectively, by neutral losses of C_6_H_12_O_2_ (116.0837 amu) and C_8_H_8_O_2_ (136.0524 amu), from molecular and/or fragment ions. Particularly, the presence of this unusual phenylacetate unit was confirmed by GC/MS analysis of FAMEs from the 90% MeOH fraction by comparing its mass spectrum with the NIST11 spectral database (Figure S7). Analysis of the ESI MS tandem spectra of the selected compounds allowed to establish unambiguously if the phenylacetate (or the hexanoate) was either directly linked to the sugar moiety, as primary acyl chain, or located at the β-hydroxy position of a certain 3-OH FA, as secondary acyl chain. Indeed, (a) the presence of fragments featuring a phenylacetate (or hexanoate) residue linked to the sugar moiety (e.g., ion **9**, Figure 10; Figures S10 and S12) and (b) the observation that a McLafferty-type rearrangement (fragmentation *e*, Figure 10) occurred only in 3-OH FAs with a free OH group (Figures S8–S12), provided useful information to assemble chemical structures as reported in Table 3.

If the presence of a hexanoate unit is widely documented, it is worth mentioning that this is the first report describing succinic saccharide esters bearing a phenylacetate moiety, to best of our knowledge. Moreover, detection of glycolipids displaying a 3-OH-4-phenylbutanoate unit further supports the biosynthetic ability of *Rhodococcus* sp. IR2 to recruit short-chain phenylalkanoic acids to assemble these novel succinic esters. Biosynthetically, the 3-OH-4-phenylbutanoic could derive either from condensation of acetate and phenylacetate units or from oxidative degradation of phenylalkanes and/or phenyl alkanoic acids [37,38] which are usually added to the bacterial growth medium. To note, the culture medium of *Rhodococcus* sp. IR2 was not supplemented with any phenylhydrocarbons.

### 2.7. Identification of Putative Genes Involved in Succinic Saccharide Esters Biosynthesis

**Figure 11:**
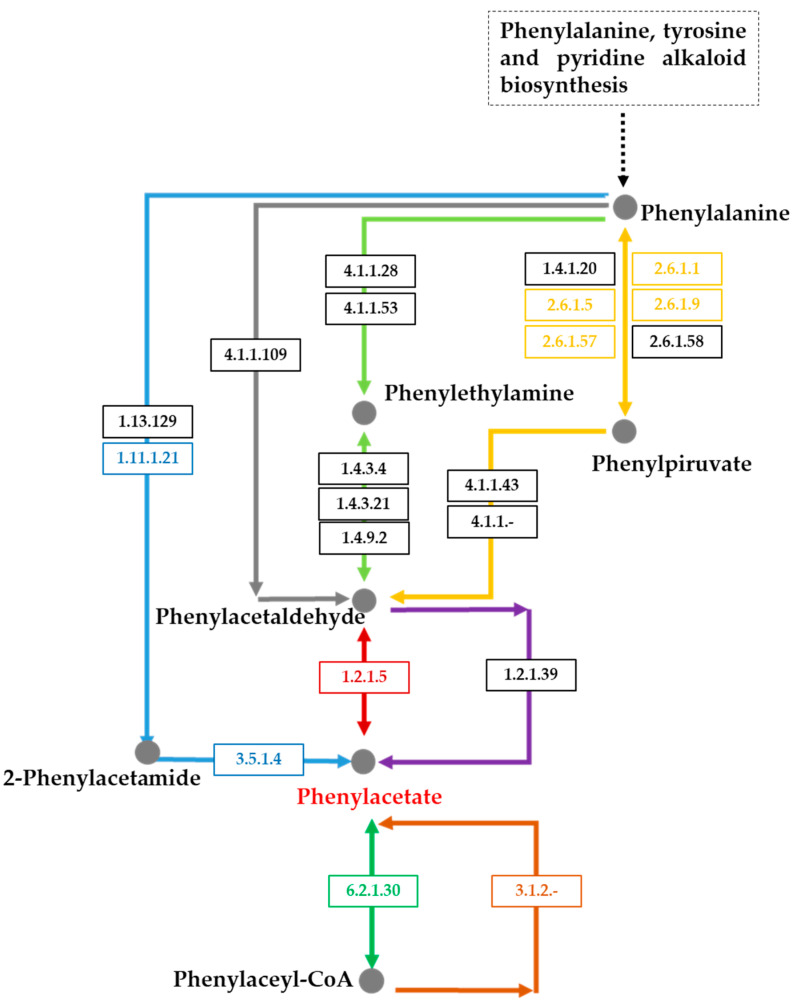
Phenylacetic acid (PhAc) biosynthesis from phenylalanine (Phe). In bacteria, PhAc is usually produced from Phe via the intermediate phenylpyruvate (PhePA). This route foresees transamination of Phe to PhePA (yellow arrow), which may undergo decarboxylation to phenylacetaldeide (PhAcld) and subsequent oxidation to PhAc (red arrow). Other biosynthetic ways, excluding formation of the intermediate PPA, foresee (a) decarboxylation of Phe to phenylethylamine (PheEA) (light green arrow), conversion to PhAcld and oxidation to PhAc, or (b) transformation into phenylacetamide (PhAcAM) (blue arrow), then converted to PhAc. Finally, enzymatic activation of PhAc to phenylacetyl-CoA is expected to be an important step to assemble succinic saccharide esters bearing a PhAc functional group. Colored boxes indicate enzymes involved in PhAc production that have been detected in the genome of *Rhodococcus* sp. I2R while dark boxes indicate enzymes that have not been identified.

The high number of novel glycolipids expressed by *Rhodococcus* sp. I2R, led to the investigation of its genome through the KBase software, searching for putative genes involved in the production of succinic saccharide esters, integrating the experimental data provided by the chemical analysis. Bioinformatic analysis (Table 5) allowed to detect the presence of trehalose-6-phosphate synthase (*otsA*) and two copies of trehalose-6-phosphate phosphatase (*otsB*) involved in de novo trehalose biosynthesis [39] and the genes malto-oligosyltrehalose synthase (*treY*), malto-oligosyltrehalose trehalohydrolase (*treZ*) and glycogen debranching enzyme (*treX*) involved in an alternative pathway for trehalose biosynthesis from maltooligosaccharides and starch or glycogen [40,41]. According to literature, two genes encoding fructose-bisphosphate aldolases, which catalyze the conversion of sugars from triose to hexose or vice versa, could play a key role in the succinic saccharide esters formation [42]. Furthermore, the genome annotation unveiled many glycosyltransferases and acyltransferases spread on several contigs, responsible for the biosynthesis of di/oligo/poly-saccharides and transfer of hydrophobic acyl groups to the sugar moiety, respectively. In particular, the contig 9 holds three copies of *papA3* genes and one *papA1*, encoding putative acyltransferases, which are reported to transfer acyl groups from CoA-donor molecules to trehalose. Moreover, contig 6 hosts a putative acyltransferase sharing approximately 42% identity (*E* value = 3 × 10^−168^) with the SucT acyltransferase from *Mycobacterium smegmatis* that adds succinyl groups to the arabinan domains of arabinogalactans and lipoarabinomannans [43]. Interestingly, this gene is adjacent to the trehalose biosynthetic genes *treZ*, *treX*, and *treY* in the genome of *Rhodococcus* sp. I2R. Several genes putatively involved in the biosynthesis of phenylacetic acid (PhAc), a characteristic moiety of our new glycolipids, have been detected. PhAc biosynthesis is included in the phenylalanine (Phe) metabolism and could follow several biosynthetic routes (Figure 11). Two putative enzymes were found, namely (1) a catalase-peroxidase 2 (EC 1.11.1.21) forming the intermediate phenylacetamide and (2) two amidases (EC 3.5.1.4), which may lead to PhAc. However, the presence of a phenylacetaldehyde dehydrogenase, which catalyzes phenylacetaldehyde oxidation to PhAc, as well as of three copies of phenylacetate-CoA oxygenase/reductase, suggest that other undescribed genes could be involved in the PhAc production.

## 3. Discussion

Marine Actinobacteria are considered a treasure for drug discovery and biotechnological industries, but the high rate of re-discovery of already known molecules represents an issue. In this work, to overcome this problem, an untargeted approach combining genomic, metabolomics and microbiology was applied to induce the production of novel bioactive molecules by the marine *Rhodococcus* sp. I2R. The taxonomic analysis demonstrated this isolate to be closely related to *Rhodococcus* sp. CUA-806 and KRD197, which have been previously isolated from marine [44] and polar [45] habitats, respectively, and reported to have high biosynthetic potential. *Rhodococcus* is a promising bacterial genus because of its metabolic versatility and ability to degrade a wide range of recalcitrant compounds and survive in highly polluted environments as recently highlighted by Cappelletti et al. [14]. Genome mining of *Rhodococcus* sp. I2R revealed the presence of 36 BGCs, around 40% of them without similarity with known genes and with a prevalence of saccharide genes (above 50% of total BGCs), indicating the production of glycosylated molecules. In the light of the biosynthetic potential of this strain, an OSMAC approach was applied to elicit the production of novel bioactive molecules. As a fact, through culture media variation (i.e., varying the concentration of carbon, nitrogen, phosphorous, inorganic salts, etc.), it is possible to upregulate or trigger the production of certain secondary metabolites produced by talented microorganisms [7,46,47,48]. In this study, the OSMAC strategy associated to bioassays allowed the selection of a specific growth condition expressing unique, structurally related metabolites, as highlighted by molecular networking (MN). MN resulted to be an effective strategy to assess the effects of different culture conditions on the microbial metabolism and shorten dereplication time [48].

Following these observations, chemical analysis of the enriched fraction F90 led to the identification of these metabolites as novel succinic saccharide esters, by integration of HR-MS/MS and GC-MS data. As often occurs for glycolipids [49], this complex mixture contained homologous compounds series and isomers and could not be separated into pure compounds. However, the F90 fraction was suitable for LC-MS studies and an in-depth analysis of HR-MS tandem spectra allowed to predict the acylation pattern of most abundant succinic di- and tri-saccharide esters. In addition to featuring succinate, the saccharide backbone was found to be acylated with hydroxylated medium-chain fatty acids, hexanoic acid, short chain phenylalkanoic acids (i.e., phenyl acetic and 3-OH-4-phenylbutanoic acids), and *O*-ester-linked acyloxyacyl motifs. As succinic saccharide esters from *Rhodococcus* spp. always have a trehalose unit and *Rhodococcus* sp. I2R possesses the biosynthetic machinery for trehalose, it could be argued these novel biosurfactants are succinoyl trehalolipids. Succinoyl trehalolipids have already been reported from other *Rhodococcus*, as well as the trisaccharo-lipids have been discovered both from *Rhodococcus* and other actinobacteria [14,29,30,31], nevertheless, to the best of our knowledge, this is the first report about the presence of phenylacetic acid as functional group of microbial glycolipids.

In general, biosurfactant molecules can be applied in various area such as the nutrient, cosmetic, textile, varnish, mining, oil recovery and pharmaceutical industries, as showing lower toxicity and improved physicochemical and functional properties in comparison to their synthetic analogues [50,51,52,53,54]. However, one of the main barriers to the biotechnological development of trehalolipids is due to the fact that they are often cell-bound, thus hampering the large-scale production and purification due to high downstream costs [13]. The ability of microbial producers to release specific glycolipids extracellularly may help to overcome this issue, thus making easier recovery of the target compounds. Examples of *Rhodococcus* strains as producers of an extracellular mixture of trehalolipids using glycerol or n-hexadecane as sole carbon source are reported [55]. Notably, in this work, the SV2 SW medium employed for cultivation of *Rhodococcus* sp. I2R stimulated the production of the glycolipid mixture in the extracellular environment, thus representing an advantage from a biotechnological perspective. SV2 SW medium (1.5% glucose, 1.5% glycerol, 1.5% peptone, 0.1% CaCO_3_ in filtered natural sea water) differentiates from the other media used in this study mainly for the relatively high concentration of nutrients, such as glycerol, glucose and peptone, which could trigger the production of the new compounds. It is widely documented that glycerol induces bacterial biosurfactants production [26,56,57,58], suggesting that this nutrient could play an important role in the production of succinic saccharide esters from *Rhodococcus* sp. I2R. However, the synergic effect of some or all of these ingredients cannot be excluded.

To date, trehalolipids have been poorly investigated as natural compounds for pharmaceutical applications [59]. Herein, we have demonstrated that the enriched fraction of succinoyl trehalolipids exerted high antiproliferative activity on a prostate cancer cell line (PC3) at 100 µg/mL while being significantly less cytotoxic to the normal counterpart (PNT2) (Figure 3). Glycolipid biosurfactants are already known to exhibit growth-inhibitory effects on many cancer cell types (e.g., SKW-3, BV-173 and HL-60) [60].

Moreover, the enriched glycolipid fraction showed a potent antiviral effect towards enveloped viruses, including herpes simplex virus type 1 and α- and β- human coronaviruses, which correlates with the observed high biosurfactant activity. Therefore, viral inhibition could be due to an interaction of the biosurfactants molecules with viral lipid envelope, as demonstrated for bacterial lipopeptides able to inactivate enveloped viruses, such as retroviruses and herpes viruses, by ion channels formation [61,62,63]. With this regard, it is worth mentioning that a succinoyl-trehalose lipid from *Rhodococcus erythropolis* has been reported to inhibit herpes simplex virus and influenza virus with LD_50_ values of 11 and 33 µg/mL, respectively [64], however, this antiviral activity was not clearly documented [64,65,66]. Moreover, it has been proven that a trehalose-6,6′-dimycolate induces resistance to influenza virus infection in mice through an augmented interferon production in T-lymphocytes [67,68]. Notably, to the best of our knowledge, this is the first manuscript describing the antiviral effects of succinoyl trehalolipids towards coronaviruses, including a β-coronavirus closely related to SARS-CoV-2.

Microbial expression of succinoyl trehalolipids is already known, however, the genetic basis and its regulation are still poorly understood. Although the elucidation of the biosynthetic pathway of glycolipids from *Rhodococcus* sp. I2R requires gene expression and functional genomics studies, a preliminary genome annotation allowed to find putative genes encoding trehalose biosynthesis [39,40,41] and acyltransferases catalyzing acylation of the sugar residue. To this date, only a putative acyl-CoA transferase, named trehalose lipid synthase A (*tlsA*), has been shown to play a key role in the addition of acyl groups to the trehalose moiety to assemble succinoyl trehalolipids from *Rhodococcus* sp. SD-74 [42]. In a Blast search, using TlsA as query sequence, four *papA* genes (clustered together in contig 9) from *Rhodococcus* sp. I2R were identified as the closest homologues, sharing approximately 30% similarity with TlsA (*E* values between 8 × 10^−70^ and 8 × 10^−30^). In addition, bioinformatic analysis shed light on potential biosynthetic routes leading to the formation of phenylacetic acid from phenylalanine, unveiling the ability of *Rhodococcus* sp. I2R to produce this unprecedented building block found in our novel succinic saccharide esters.

## 4. Materials and Methods

### 4.1. Isolation, Identification, and Genome Sequencing of Strain I2R

The strain I2R was isolated on agar plates containing Mineral Salt Medium (MSM) supplemented with 1.0 mM of phenanthrene from marine sediments collected during the oceanographic campaign “Earth Cruisers 18” in the Southern Tyrrhenian Sea in February 2018. The phylogenetic affiliation of the bacterium was performed through the 16S rRNA genes amplification and analysis. The bacterial genomic DNA was isolated using “GeneElute Bacterial genomic DNA kit” according to the manufacturer instructions (Sigma-Aldrich, Darmstadt, Germany) from a 2 mL I2R culture in TYP and used as template for the PCR.

PCR was carried out in 50 µL reaction containing DreamTaq PCR Master Mix (a ready-to-use solution containing DreamTaq DNA Polymerase, optimized DreamTaq buffer, MgCl_2_, and dNTPs) and 0.2 µM of primer Eub27 F (Forward, seq: 50-AGAGTTTGATCCTGGCTCAG-30) and Univ1492R (Reverse, seq: 50-GGTTACCTTGTTACGACTT-30) [69]. The reaction conditions used were: one cycle (95 °C for 2 min), 30 cycles (95 °C for 30 s, 55 °C for 30 s, and 72 °C for 1 min), with a final extension of 5 min at 72 °C. PCR products were then purified by GenEluteTM PCR Clean-UP kit (Sigma-Aldrich, Darmstadt, Germany), the purified PCR products were sequenced by Eurofins Genomics (Ebersberg, Germany). Both end sequences obtained by submitting the forward and the reverse to Prabi CAP3 [70] (http://doua.prabi.fr/software/cap3, accessed on 1 July 2020) were submitted to BLAST for preliminary phylogenetic analysis.

A total of 1 µg of genomic DNA was used for the whole genome sequencing, performed by Macrogen Europe (Amsterdam, The Netherlands) using the Illumina NovaSeq 6000 platform, with a read length of 151 bp. The obtained 16,283,548 raw reads were trimmed through Trimmomatic tool (v0.36) [71] (http://www.usadellab.org/cms/index.php?page=trimmomatic, accessed on (20 August 2020) to remove adaptor and low-quality sequences, and a quality check was obtained with FastQC (v0.11.5) [72] (https://www.bioinformatics.babraham.ac.uk/projects/fastqc/, accessed on 1 September 2020.

Finally, after a K-mer analysis through Jellyfish (v2.2.10) [73] (http://www.genome.umd.edu/jellyfish.html, accessed on 1 September 2020) to estimate the genome length, the de novo assembly was performed using SPAdes [74] (https://cab.spbu.ru/software/spades/, accessed on 5 September 2020) and the completeness of the genome was assessed by BUSCO (v3.0.2) analysis [75] (https://busco.ezlab.org/, accessed on 5 September 2020).

Genome-based identification was carried out using ANI Calculator by EZBiocloud [76]. The sequence of *Rhodococcus* sp. I2R generated in this study have been deposited in the GenBank database under the accession number JAHUTG000000000 for bacterial whole genome genes.

### 4.2. Genome Annotation

The obtained genome was automatically annotated using both RASTtk (v1.073) [17] and PROKKA (v1.14.5) [77] by Kbase [78]. The metabolomic pathways were reconstructed performing KEGG BlastKOALA and KEGG mapping [79] analysis, filling the gaps manually. Finally, antiSMASH 6.0 (detection strictness: loose) [80] was applied to detect and annotate the biosynthetic gene clusters (BGCs) contained in the whole genome sequence.

### 4.3. OSMAC Approach and Growth Media

A single colony of strain I2R was used to inoculate 10 mL of liquid medium TYP in 50 mL conical tubes and incubated for 48 h at 20 °C at 180 rpm. After the incubation, this pre-inoculum was centrifuged at 5000× *g* at 4 °C for 20′, the supernatant discarded and the pellet washed twice with 10 mL of sterile water to eliminate residues of the TYP medium and centrifuged. Finally, the pellet was resuspended with 5 mL of sterile water and 200 µL were used to inoculate 22 flasks (100 mL volume) containing 20 mL of the growth media listed below. Cultures were incubated for 6 days at 20 °C at 180 rpm. The list of growth media used for the OSMAC approach is detailed below. Filtered sea water (SW), autoclaved distilled water and MSM (KH_2_PO_4_ 0.7 g/L, Na2HPO_4_ 0.9 g/L, NaNO_3_ 2 g/L, MgSO_4_ 0.4 g/L, CaCl_2_ 0.1 g/L) have been used to prepare the growth media:

**AUR SW:** starch 10 g/L, glucose 10 g/L, glycerol 10 g/L, peptone 5 g/L, yeast extract 2 g/L, in sea water.

**GYM SW:** glucose 4 g/L, yeast extract 4 g/L, malt extract 4 g/L, CaCO_3_ 2 g/L, in sea water.

**LAR MSM:** mannitol 30 g/L, glucose 10 g/L, yeast extract 5 g/L, ammonium succinate 5 g/L, in MSM.

**LB:** tryptone 10 g/L, yeast extract 5 g/L, NaCl 10 g/L, in distilled water.

**NOS SW:** glucose 20 g/L, yeast extract 3 g/L, peptone 10 g/L, malt extract 15 g/L, Maltose 20 g/L, in sea water.

**MSM Gly:** glycerol 10 g/L, in MSM.

**MSM Glu Cas:** glucose 10 g/L, casein hydrolyzed 15 g/L, in MSM.

**SV2 SW:** glucose 15 g/L, peptone 15 g/L, glycerol 15 g/L, CaCO_3_ 1 g/L, in sea water.

**TGB:** thioglycollate broth with resazurine (Condalab, Madrid, Spain) in distilled water.

**MB:** marine broth (Condalab, Madrid, Spain) in distilled water.

**PM2 SW:** starch 10 g/L, yeast extract 4 g/L, peptone 2 g/L, Fe_2_(SO_4_)_3_ × 4H_2_O 40 mg/mL, in sea water.

**MSM Pep Glu:** glucose 10 g/L, peptone 10 g/L, in MSM.

**MSM Glu Arg:** glucose 10 g/L, arginine 30 mM, in MSM.

**MSM Gly Arg:** glycerol 10 g/L, arginine 30 mM, in MSM.

**TYP:** tryptone 6 g/L, yeast extract 16 g/L, NaCl 10 g/L, in distilled water.

**ISP2 MSM:** glucose 4 g/L, yeast extract 4 g/L, malt extract 10 g/L, in MSM.

**ISP2 SW:** glucose 4 g/L, yeast extract 4 g/L, malt extract 10 g/L, in sea water.

**PPGAS:** glucose 5 g/L, peptone 10 g/L, NH_4_Cl 20 mM, KCl 20 mM, Tris-HCl (pH 7.2) 120 mM, MgSO_4_ 1.6 mM, in distilled water.

**MSM Glu:** glucose 10 g/L in MSM.

**QUIN MSM:** fructose 2 g/L, casein hydrolyzed 2 g/L, in MSM.

**MSM Glu Urea:** glucose 10 g/L, urea 2 g/L, in MSM.

**TSB:** tryptone soy broth (Condalab, Madrid, Spain) in distilled water.

### 4.4. Preparation of Crude Extracts

After 6 days, the 22 cultures were centrifuged at 6800× *g* at 4 °C for 30′. The bacterial pellet was stored at −20 °C for further studies, while the exhausted culture broths were subjected to organic extraction. One volume of broth was mixed with two volumes of ethyl acetate in 250 mL separator funnels. The organic phase was collected and evaporated using a rotavapor (R-100, BUCHI, Flawil, Switzerland) and the extracts were weighted and split for different uses as follow: 1 mg of the extract amount was dissolved in 1 mL of mass grade MeOH for mass spectrometry analyses, while the remaining extract was dissolved in 100% DMSO at the concentration 25 mg/mL and stored at −20 °C to be used for functional assays.

### 4.5. Bioactivity Evaluation by Functional Assays

A wide range of functional assays has been carried out to evaluate the biological effect of the 22 crude extracts.

#### 4.5.1. Antiviral Assays

##### Virus, Cell Culture and Treatment

Vero cells ATCC CCL-81, kidney epithelial cell line from African green monkey (*Cercopithecus aethiops)* were purchased from American Type Culture Collection (ATCC, Manassas, VI, USA). Vero cells were grown in Dulbecco’s Modified Eagle Medium (DMEM) (Microtech, Naples, Italy) supplemented with 10% fetal bovine serum (FBS) (Microtech, Naples, Italy), 100 mg/mL of streptomycin, and 100 IU/mL of penicillin in a humidified atmosphere with 5% CO2 at 37 °C. Herpes simplex virus type-1 strain SC16 (HSV-1), Green Fluorescent Protein herpes simplex virus type 1 (HSV-1-GFP) [81], human coronavirus 229E ATCC VR-740 (HCoV-229E), human coronavirus OC43 ATCC VR-1558 (HCoV-OC43), and Poliovirus Type 1, strain Chat ATCC VR-1562 (PV-1) were purchased from ATCC and propagated on Vero cells (Table 6). The cell viability was determined through colorimetric MTT assay (see Section 4.5.3).

##### HSV-1 Co-Treatment Assay

To evaluate the effect of the different samples on HSV-1 infectivity inhibition, a co-treatment experiment was performed. Vero cells were plated into 12-well cell culture plates (2 × 10^5^ cells for well) in culture medium. The next day, the extracts were dissolved in DMEM at different concentrations, from 125 to 500 µg/mL, in the presence of HSV-1 at 10^3^ plaque forming units (PFU) and dispensed on the cells in free FBS medium for 1 h at 37 °C. At the end of the treatment, the cell monolayer was washed with Phosphate Buffer Saline (PBS) (Microtech, Naples, Italy) and incubated in a fresh culture medium supplemented with 5% carboxymethylcellulose (CMC) (Sigma-Aldrich, Darmstadt, Germany) for 48 h. After the incubation, the cells were fixed with 4% formaldehyde and stained with 1% crystal-violet, and the plaques were counted. The percentage of viral inhibition was calculated by counting the number of plaques obtained in presence of extract with respect to the untreated virus (CTRL–). Greco extract at 50 µg/mL was used as positive control (CTRL+) [25].

##### Virus Pre-Treatment Assay

To evaluate the effect of the fractions on HSV-1, HSV-1-GFP, HCoV-229E, and Poliovirus PV-1, a virus pre-treatment experiment was performed. Vero were plated into 12-well (2 × 10^5^ cells/well) in culture medium and incubated for 24 h in a humidified atmosphere. The fractions were added at different concentrations (from 0.4 to 500 μg/mL) to a viral suspension in DMEM containing 1 × 10^4^ PFU, and incubated for 1 h at 37 °C. After incubation, each mixture was diluted in FBS free DMEM and titrated on Vero cells for 1 h. Later, the cells were washed with PBS 1X and incubated for 24 h (HCoV-229E) or 48 h (HSV-1, HSV1-GFP and PV-1) in DMEM supplemented with CMC.

After 2 days, the cells were fixed and stained with 1% crystal-violet, and the plaques were counted. Differently, after the incubation, HSV-1-GFP was not further processed, images were acquired through the Nikon ECLIPSE Ti2-U inverted florescence microscope (Nikon Europe B.V., Amsterdam, Netherlands) with beam settings for FITC and BF. The negative control is represented by the cells treated only with the virus (without sample). Pleconaril [82] at 1 µM was used as positive control against PV-1, while greco extract at 50 µg/mL was used as positive control (CTRL+) towards the other viruses.

##### Antiviral Assay against Human Coronavirus (HCoV-OC43)

To verify the antiviral action against HCoV-OC43, the inhibition of the cytopathic effect induced by the virus on the Vero cells was evaluated. Briefly, 2 × 10^4^ Vero cells/well were seeded in 96-well plates and incubated for 24 h at 37 °C in a humidified CO_2_ (5%) atmosphere. Cell monolayers were then infected with 100 μL of a proper virus dilution in a maintenance medium. At the same time, 100 μL of medium, without or with serial dilutions of the tested compounds, were added. Greco extract at 50 µg/mL was used as positive control. After 3 days of incubation at 37 °C, cell viability was determined by the MTT assay (see Section 4.5.3).

#### 4.5.2. Antiproliferative Assays

##### Maintenance of Human Cell Cultures

The human prostatic adenocarcinoma cell line (PC3) and the human prostatic epithelial cell line (PNT2) were maintained using RPMI 1640 medium supplemented with 10% (*v*/*v*) fetal bovine serum (FBS), 2 mM L-glutamine, 100 units/mL penicillin and 100 μg/mL streptomycin. Cells were grown in a 5% CO2 atmosphere at 37 °C, maintaining confluence under 70%.

##### Cytotoxicity Assay

Cells (2 × 10^3^ cells/well, 100 µL final volume) were seeded in 96-well plates and kept overnight for attachment for cytotoxicity assessment. DMSO was used at a final concentration of ≤0.5% (*v*/*v*) for each treatment. Cells were treated in biological triplicate with 1, 10, and 100 µg/mL of extracts for 48 h in a complete cell medium. At the end of incubation with the samples, cytotoxicity was evaluated by MTT assay (see Section 4.5.3). Untreated cells were used as negative control.

#### 4.5.3. MTT Assay

MTT assay was used to assess cell viability after antiproliferative and antiviral treatments using the Thiazolyl Blue Tetrazolium Bromide [3-(4,5-dimethylthiazol-2-yl)-2,5-diphenyltetrazolium bromide] (Sigma-Aldrich, Darmstadt, Germany). After treatment, the supernatant was removed and 0.5 mg/mL of MTT water solution was added to the cells for 3 h. After emptying the plate, 100 µL of DMSO were added in each well to solubilize the formazan crystals. Finally, the absorbance was read at 570 nm with a microplate reader. The control is represented by untreated cells.

#### 4.5.4. Biosurfactant Screening Assay by CTAB Agar Method

The CTAB agar method, also called Blue agar, is an in-plate test that can reveal the presence of anionic biosurfactants by the arising of dark blue halos around the extracts. In this method, the anionic biosurfactants form an insoluble complex with cetyltrimethylammonium bromide, and the complex is revealed by the presence of methylene blue. 4 µL of the extracts, dissolved in DMSO at 25 mg/mL were spotted on the blue agar plates. As a negative control, 8 µL of pure DMSO were used, while 4 µL of 0.1% and 0.01% sodium dodecyl sulphate (SDS) were used as positive controls. After 2 days at 4 °C, the extracts containing biosurfactants were selected by the presence of a dark blue halo around the wells.

### 4.6. Chemical Profiling by Mass Spectrometry and Molecular Networking

The extracts obtained in the 22 different media were dissolved in mass grade MeOH and subjected to a liquid chromatography-mass-spectrometry analysis by performing a low-resolution mass spectrometry (LRMS) data-dependent analysis. The chromatographic separation was achieved on a Synergi 2.5 mm Fusion_RP 100×2 by using a 20 min linear gradient from 0% to 100% of buffer B (Buffer A: H2O + 0.1% Formic acid, Buffer B: ACN + 0.1% Formic acid). Mass spectra were recorded in positive and negative mode within a mass range of 150–1500 Da, and the most abundant ions were subjected to MS/MS fragmentation with a rolling collision energy between 0–80 eV.

Low-resolution MS/MS data from the 22 crude extracts were converted to *.mzXML by using the tool msconvert from ProteoWizard [83] and imported into MZmine 2.53. Mass detection was performed on .mzXML data and centroided masses with mass level 1 and mass level 2, by keeping the noise level at 1000 and 100, respectively. Chromatograms were built using the ADAP chromatogram algorithm [84] with a minimum height of 1000, and *m/z* tolerance of 1. For chromatogram deconvolution, the baseline cut-off algorithm was employed with the following settings: minimum height peak = 1000, peak duration range = 0.0–3.0 min, baseline level = 100, *m*/*z* range for MS2 scan = 1, retention time range = 0.15 min. [M+1, ^13^C] adducts were filtered out using the “isotopic peaks grouper” module, by setting *m*/*z* tolerance at 0.1 and and *R_t_* tolerance at 0.1 min. Peak alignment was performed using the Join aligner algorithm (*m/z* tolerance at 0.5, absolute *R_t_* tolerance at 0.6 min). Peaks without associated MS/MS spectrum were finally filtered out from the peak list. Clustered data were then exported to .mgf file for GNPS, while chromatographic information of each peak was exported to a quantification table as .csv format file. Processed data were submitted to GNPS [8] to create a molecular network by using the Feature Based Molecular Networking (FBMN) tool [28]. FBMN parameters were set as follows: precursor ion mass and fragment ion mass tolerances = 0.5 Da, cosine score ≥ 0.7, minimum matched fragment ions = 4. The molecular network (available at https://gnps.ucsd.edu/ProteoSAFe/status.jsp?task=c8ae043f75914cbcadf441d283670291, accessed on 1 April 2021) was visualized and analyzed in Cytoscape 3.7.2. Chromatographic data in the .csv file were mapped to the relevant nodes in the generated network.

### 4.7. Scale-Up Fermentation of I2R in SV2 SW Medium and Crude Extract Fractionation

*Rhodococcus* sp. I2R was grown in 400 mL of SV2 SW medium for 6 days at 20 °C; then, the culture broth was extracted with 2 volumes of ethyl acetate. Subsequently, the crude extract was fractionated with a Chromabond SPE C18 column Cartridge (Macherey-Nagel, Duren, Germany). Elution was performed stepwise with an increasing methanol concentration. The gradient was selected based on the spectra derived from mass spectrometry analysis. Gradient: H_2_O, 50% MeOH, 90% MeOH, 100% MeOH. The three eluted fractions (water excluded) were collected, dried and dissolved in DMSO at stock concentration of 25 mg/mL to perform the antiviral, antiproliferative, and biosurfactant assay.

### 4.8. Data-Dependent LC-HRMS/MS Analysis of the 90% MeOH Fraction

The 90% MeOH from the crude extract of *Rhodococcus* sp. I2R cultivated in SV2 SW was dried under vacuum and dissolved in MeOH at a concentration of 1 mg/mL for LC-HRMS/MS (Liquid Chromatography—High Resolution Tandem Mass Spectrometry) analyses, as previously reported [85]. Experiments were performed using a Thermo LTQ Orbitrap XL high-resolution ESI mass spectrometer coupled to a Thermo U3000 HPLC system, which included a solvent reservoir, in-line degasser, binary pump, and refrigerated autosampler. A 5-μm Kinetex C18 column (50 × 2.10 mm), maintained at room temperature, was eluted at 200 μL·min^−1^ with H_2_O (supplemented with 0.1% HCOOH) and CH_3_CN, using a gradient elution. The gradient program was set as follows: 5% CH_3_CN 3 min, 5–99% CH_3_CN over 30 min, 100% CH_3_CN 7 min. A survey full MS scan was acquired in positive and negative ion detection modes. MS parameters were a spray voltage of 4.8 kV, a capillary temperature of 285 °C, a sheath gas rate of 32 units N_2_ (ca. 150 mL/min), and an auxiliary gas rate of 15 units N_2_ (ca. 50 mL/min). Data were collected in the data-dependent acquisition mode to perform high resolution tandem mass spectrometry (HRMS/MS) analysis of selected ions reported in Table 1, in negative ion detection mode. The *m/z* range for data-dependent acquisition was set between 100 and 2000 amu. HRMS/MS scans were obtained for selected ions with CID fragmentation, an isolation width of 2.0, normalized collision energy of 35, activation Q of 0.250, and an activation time of 30 ms.

### 4.9. Methanolysis of the 90% MeOH Fraction and GC-MS Analysis of FAMEs

The 90% MeOH fraction (800 µg) from crude extract SV2 SW, was dissolved in 6 M HCl (500 µL) and kept at 90 °C for 16 h. The hydrolysis product was dried under nitrogen, then 500 µL CHCl_3_, 450 µL MeOH, and 75 µL H_2_SO_4_ were added and the reaction mixture was incubated again at 90 °C for 16 h to yield fatty acid methyl esters (FAMEs), according to a slightly modified protocol reported in the literature [86]. After cooling to room temperature, an equal volume of distilled water was added, and then time was allowed for phase separation. The lower phase, which contained FAMEs, was collected and dried under nitrogen. FAMEs were analyzed by GC/MS on a 5390 MSD quadrupole mass spectrometer (Agilent, Cernusco sul Naviglio, Milano, Italy), using an HP-5MS capillary column (Agilent, 5% Phenyl Methyl Siloxane) (30 m length, 0.25 mm Ø, 0.25 μm film). Helium was used as a carrier gas, injection was in split mode, and the program was as follows: hold 50 °C for 1 min, heat to 140 °C with 8 °C/min, hold 140 °C for 2 min, heat to 180 °C with 5 °C/min, heat to 280 °C with 10 °C/min, hold 280 °C for 5 min.

### 4.10. Statistical Analysis

Bioassay data represent the mean (±standard deviation, SD) of at least three independent experiments. Differences between groups were determined by analysis of variance (ANOVA) and were considered statistically significant at *p* < 0.05. Statistical analysis was performed using the GraphPad Prism Software Version 8 (GraphPad Software Inc., San Diego, CA, USA).

## 5. Conclusions

The marine environment is a promising source for a large variety of surface-active metabolites. Undoubtedly, their chemical diversity is much larger than described until today and the structures of many biosurfactants still remain unknown. Herein, once again, Actinobacteria clearly have been shown to be a unique and vast untapped resource for the discovery of novel and potentially useful biosurfactants. The application of an OSMAC approach, combined with a highly metabolically versatile bacterium and the use of LC-HRMS/MS, allowed the production and identification of a complex extracellular mixture of novel succinic saccharide esters with an uncommon functional group, i.e., phenylacetic acid. For the first time, we observed the antiviral effects of these glycolipid molecules against coronaviruses, leading to virus inactivation likely through a detergent-like mechanism. Due to the high complexity of the active fraction, the antiviral assays were carried out on the mixture. Future perspectives will include (a) optimization of culture conditions to favor the production of a specific class of biosurfactants, (b) purification of single molecules for NMR stereo-structural elucidation, and (c) characterization of the biosynthetic pathways for succinoyl saccharide esters through transcriptomic analysis and functional genomics studies.

## Figures and Tables

**Table 1:** General features of the genome of *Rhodococcus* sp. I2R.

Attribute	Value
Genome size (bp)	5,290,284
DNA G+C (%)	64.01
Number of contigs	72
Longest contig length (bp)	336,301
Shortest contig length (bp)	1117
Average contig length (bp)	73,476
N50 (bp)	195,979
Number of Coding Sequences	5210
RNA genes (tRNA + rRNA)	52

**Table 2:** antiSMASH identification and analysis of BGCs in *Rhodococcus* sp. I2R whole genome sequence.

Type	Contig	Location (nt)	Most Similar Known Cluster	Similarity (%)
Saccharide—NRPS	1	29,248–91,486	Coelichelin	27
Saccharide	1	313,381–336,301	Macrotetrolide	33
T2PKS—saccharide	2	47,197–119,676	Mayamycin	63
Saccharide	2	236,836–273,343	TP-1161	8
NRPS—redox-cofactor—saccharide	3	1–92,466	–	–
Ectoine	4	116,604–127,002	Ectoine	50
Saccharide—T1PKS	5	1–59,796	Selvamicin	11
Fatty acid	5	148,163–167,687	–	–
Fatty acid	5	212,303–233,406	–	–
Saccharide	6	107,905–134,028	–	–
Saccharide—terpene	6	144,642–204,805	Isorenieratene	42
Saccharide	7	153–31,205	–	–
Saccharide	7	42,961–63,566	Tetronasin	3
Saccharide	7	96,022–137,264	Rimosamide	14
NRPS-like	7	142,446–185,022	–	–
Terpene	8	92,586–113,764	SF2575	6
Saccharide	9	108,630–145,274	ECO-02301	7
Saccharide	10	73,245–94,928	Streptovaricin	4
Saccharide	10	98,951–137,834	–	–
PKS-like—amglyccycl *	12	158,941–192,419	–	–
Furan—fatty acid	13	13,932–35,940	Diisonitrile antibiotic SF2768	11
NAPAA *	15	1–30,389	–	–
hglE-KS *	16	63,473–105,151	Vazabitide A	10
NRPS	17	1–29,241	Atratumycin	5
NRPS—saccharide	18	1–79,116	–	–
Butyrolactone	21	68,428–79,324	–	–
Arylpolyene—T1PKS—fatty acid—PKS-like	24	1–67,243	Abyssomicin C/Atrop-Abyssomicin C	7
NRPS—saccharide	26	1–59,270	Heterobactin A/Heterobactin S2	63
NRPS	27	1–54,662	Siamycin I	8
Fatty acid—saccharide	28	3562–54,528	Bottromycin A2	9
NRPS-like	29	27,485–54,026	–	–
Saccharide	32	1–17,712	–	–
Saccharide	32	34,798–52,216	Tetrocarcin A	4
NRPS	39	1–22,870	–	–
NRPS	43	1–12,753	Atratumycin	5
Saccharide	54	1–5008	–	–

* *Abbreviations*: *amglyccycl*, aminoglycoside/aminocyclitol cluster; *NAPAA*, non-alpha poly-amino acids like e-Polylysin; *hglE-KS*, heterocyst glycolipid synthase-like PKS.

**Table 3:** Succinoyl saccharide esters from *Rhodococcus* sp. I2R.

	*R*_t_ (min.)	[M − H]^–^	*m*/*z*	Primary Acyl Chains				Secondary Acyl Chains ^a^	
**disaccharide** **succinic diesters**	14.6	C_26_H_43_O_16_	611.2546	3-OH-C10				-	
	15.4	C_25_H_41_O_15_	581.2424	C9				-	
	16	C_27_H_45_O_16_	625.2685	3-OH-C11				-	
	16.3	C_26_H_41_O_15_	593.2427	C10:1				-	
	16.7	C_26_H_43_O_15_	595.2585	C10				-	
	18	C_32_H_45_O_17_	701.2633	3-OH-C8 ^b^				PhAc	
	19.3	C_30_H_51_O_16_	667.3155	3-OH-C14				-	
	19.9	C_34_H_49_O_17_	729.2948	3-OH-C10				PhAc	
**disaccharide** **succinic triesters**	16.6	C_34_H_49_O_18_	745.2901	3-OH-C8		3-OH-PhBu		-	
	16.9	C_32_H_45_O_17_	701.2634	3-OH-C8		PhAc		-	
	17.5	C_32_H_53_O_18_	725.3209	3-OH-C8		3-OH-C8		-	
	17.8	C_40_H_59_O_20_	859.3571	3-OH-C8		diOH-C8		PhAc	
	18.5	C_33_H_55_O_18_	739.3367	3-OH-C9		3-OH-C8		-	
	19	C_34_H_49_O_17_	729.2944	3-OH-C10		PhAc		-	
	19.4	C_34_H_57_O_18_	753.3523	3-OH-C10		3-OH-C8		-	
	19.8	C_38_H_55_O_19_	815.3307	3-OH-C8		3-OH-C6		PhAc ^c^	
	20.8	C_39_H_57_O_19_	829.3463	3-OH-C8		3-OH-C7		PhAc ^c^	
	20.9	C_42_H_55_O_19_	863.3309	3-OH-C8		3-OH-PhBu		PhAc	
	21.6	C_40_H_59_O_19_	843.3619	3-OH-C8		3-OH-C8		PhAc	
	22.4	C_41_H_61_O_19_	857.3775	3-OH-C9		3-OH-C8		PhAc ^c^	
	25.2	C_44_H_67_O_19_	899.4243	3-OH-C10		3-OH-C10		PhAc	
**trisaccharide** **succinic diesters**	17.2	C_38_H_55_O_22_	863.3155	3-OH-C8				PhAc	
**trisaccharide** **succinic triesters**	18	C_40_H_67_O_23_	915.4041	3-OH-C10		3-OH-C8		-	
	18.4	C_48_H_73_O_25_	1049.4406	3-OH-C10		diOH-C8		PhAc	
	19.5	C_48_H_65_O_24_	1025.3827	3-OH-C8		3-OH-PhBu		PhAc	
	20.1	C_44_H_73_O_24_	985.4456	3-OH-C8		3-OH-C8		C6	
	20.1	C_46_H_69_O_24_	1005.4138	3-OH-C8		3-OH-C8		PhAc	
	20.8	C_47_H_71_O_24_	1019.4294	3-OH-C9		3-OH-C8		PhAc ^c^	
	21.5	C_48_H_73_O_24_	1033.4447	3-OH-C10		3-OH-C8		PhAc ^c^	
	22.2	C_49_H_75_O_24_	1047.4602	3-OH-C10		3-OH-C9		PhAc	
	22.2	C_49_H_75_O_24_	1047.4602	3-OH-C11		3-OH-C8		PhAc	
	23.1	C_50_H_77_O_24_	1061.4761	3-OH-C10		3-OH-C10		PhAc	
	23.6	C_55_H_77_O_25_	1137.4707	3-OH-C9		3-OH-C8		PhAc	PhAc ^d^
	23.7	C_52_H_79_O_25_	1103.4865	3-OH-C8		3-OH-C8		PhAc	C6 ^d^
	24.6	C_53_H_81_O_25_	1117.5018	3-OH-C9		3-OH-C8		PhAc	C6 ^d^
	26.3	C_55_H_85_O_25_	1145.53296	3-OH-C11		3-OH-C8		PhAc	C6 ^d^
	26.3	C_55_H_85_O_25_	1145.53296	3-OH-C10		3-OH-C9		PhAc	C6 ^d^
**trisaccharide** **succinic tetraesters**	27.9	C_54_H_83_O_24_	1115.5221	3-OH-C8	C10		C6 ^e^	PhAc	
	29.4	C_58_H_89_O_26_	1201.5579	3-OH-C8	3-OH-C8		C6 ^e^	PhAc	C6 ^d^

*Abbreviations*: *PhAc*, phenyl acetate; *3-OH-PhBu*, 3-OH-4-phenylbutanoate. ^a^ secondary acyl chains linked to 3-OH-fatty acids as indicated in Figure 9. ^b^ 3-OH FAs with OH highlighted in red bear a secondary acyl chain through *O*-ester linkage. ^c^ mixture of isomers differing for the position of the secondary acyl chain which can be linked either to 1st or 2nd primary acyl chain. ^d^ compounds displaying two secondary acyl chains on the 1st and 2nd primary acyl chains, respectively. ^e^ compounds featuring a third sugar unit, which bears a hexanoate unit (C6).

**Table 4:** 3-hydroxy fatty acyl composition of the 90% MeOH fraction from crude extract I2R SV2 SW.

3-Hydroxy FAMEs	Relative Abundance (%)
3-OH-C6	0.2
3-OH-C7, branched	0.1
3-OH-C7	0.5
3-OH-C8, branched	6.0
3-OH-C8	31.7
3-OH-C9, branched	0.7
3-OH-C9	8.0
3-OH-C10, branched	3.0
3-OH-C10	36.1
3-OH-4-phenylbutanoate	2.2
3-OH-C11	2.8
3-OH-C12	7.6
3-OH-C13	0.2
3-OH-C14	1.0

**Table 5:** Putative genes involved in the biosynthesis of the novel succinic saccharide esters.

Contig	Start	Strand	Length	Putative Gene	Function
contig4	178,279	−	2043	−	Trehalase
contig6	117,624	+	2274	*treX*	Glycogen debranching enzyme
contig6	119,901	+	2418	*treY*	Putative maltooligosyl trehalose synthase
contig6	122,315	+	1740	*treZ*	Malto-oligosyltrehalose trehalohydrolase
contig15	69,438	−	2550	*otsB*	Trehalose-6-phosphate phosphatase
contig18	99,608	−	2181	*treS*	Trehalose synthase
contig19	37,715	+	3192	*otsB*	Trehalose-6-phosphate phosphatase
contig23	53,441	+	1458	*otsA*	Trehalose-6-phosphate synthase
contig44	6907	+	1068	*fbaA*	Fructose-bisphosphate aldolase
contig44	9581	+	1041	*fbaA*	Fructose-bisphosphate aldolase
contig6	115,388	+	2202	*sucT*	Putative succinoyl transferase
contig5	46,744	−	1887	−	Trehalose O-mycolyltransferase
contig9	137,187	+	1461	*papA3*	Acyltransferase papA3
contig9	138,665	+	1431	*papA1*	SL659 acyltransferase papA1
contig9	140,099	+	1404	*papA3*	Acyltransferase papA3
contig9	143,010	+	1488	*papA3*	Acyltransferase papA3
contig2	114,384	+	1071	*paaK*	Phenylacetate-CoA oxygenase/reductase, PaaK subunit
contig3	53,842	+	1044	*paaK*	Phenylacetate-CoA oxygenase/reductase, PaaK subunit
contig3	219,048	+	1449	*feaB*	Phenylacetaldehyde dehydrogenase
contig4	26,516	−	2238	*katG*	Catalase-peroxidase 2
contig4	233,312	−	204	*paaK*	Phenylacetate-CoA oxygenase/reductase, PaaK subunit
contig14	61,296	+	795	*amiE*	Aliphatic amidase AmiE
contig20	84,586	−	840	*amiE*	Aliphatic amidase AmiE
contig7	14,325	+	3195	*mmpL3*	Trehalose monomycolate exporter MmpL3
contig10	71,540	−	1371	*lpqY*	Trehalose-binding lipoprotein LpqY
contig12	94,464	+	2226	*mmpL3*	Trehalose monomycolate exporter MmpL3
contig17	98,444	−	2136	*mmpL3*	Trehalose monomycolate exporter MmpL3
contig5	96,103	+	1053	*sugC*	Trehalose import ATP-binding protein SugC
contig5	265,744	+	1050	*sugA*	Trehalose transport system permease protein SugA
contig5	266,790	+	822	sugB	Trehalose transport system permease protein SugB
contig7	42,107	−	1101	*sugC*	Trehalose import ATP-binding protein SugC
contig7	42,961	−	840	*sugB*	Trehalose transport system permease protein SugB
contig7	43,911	−	951	*sugA*	Trehalose transport system permease protein SugA
contig7	56,201	+	948	*sugA*	Trehalose transport system permease protein SugA
contig7	100,213	−	1083	*sugC*	Trehalose import ATP-binding protein SugC
contig10	68,393	−	1170	*sugC*	Trehalose import ATP-binding protein SugC
contig10	69,232	−	834	*sugB*	Trehalose transport system permease protein SugB
contig10	70,173	−	942	*sugA*	Trehalose transport system permease protein SugA
contig13	120,539	−	1077	*sugC*	Trehalose import ATP-binding protein SugC
contig20	57,846	−	1149	*sugC*	Trehalose import ATP-binding protein SugC
contig20	59,851	−	867	*sugB*	Trehalose transport system permease protein SugB
contig20	60,832	−	978	*sugA*	Trehalose transport system permease protein SugA

**Table 6:** Main characteristics of the viruses utilized in this work.

Virus	Family	Nucleic Acid	Symmetry	Envelope	Dimensions
HSV-1/GFP	*Herpesviridae*	dsDNA	icosahedral	yes	155–240 nm
HCoV-229E	*Coronaviridae*	ssRNA(+)	helical	yes	80–120 nm
HCoV-OC43	*Coronoviridae*	ssRNA(+)	helical	yes	80–120 nm
PV-1	*Picornaviridae*	ssRNA(+)	icosahedral	no	30 nm

ds: double strand; ss: single strand; (+): positive strand.

## Data Availability

Not applicable.

## Supplementary Materials

The following are available online at www.mdpi.com/article/10.3390/ijms22169055/s1.
