# Supplementary material for: Combining OSMAC Approach and Untargeted Metabolomics for the Identification of New Glycolipids with Potent Antiviral Activity Produced by a Marine Rhodococcus
Source: Int J Mol Sci. 2021 Aug 22;22(16):9055. doi: 10.3390/ijms22169055 (PMC8396431; doi:10.3390/ijms22169055)
Supplement: Supplementary file 1 [file ijms-22-09055-s001.zip › ijms-1325780-supplementary.pdf]

Supplementary Material

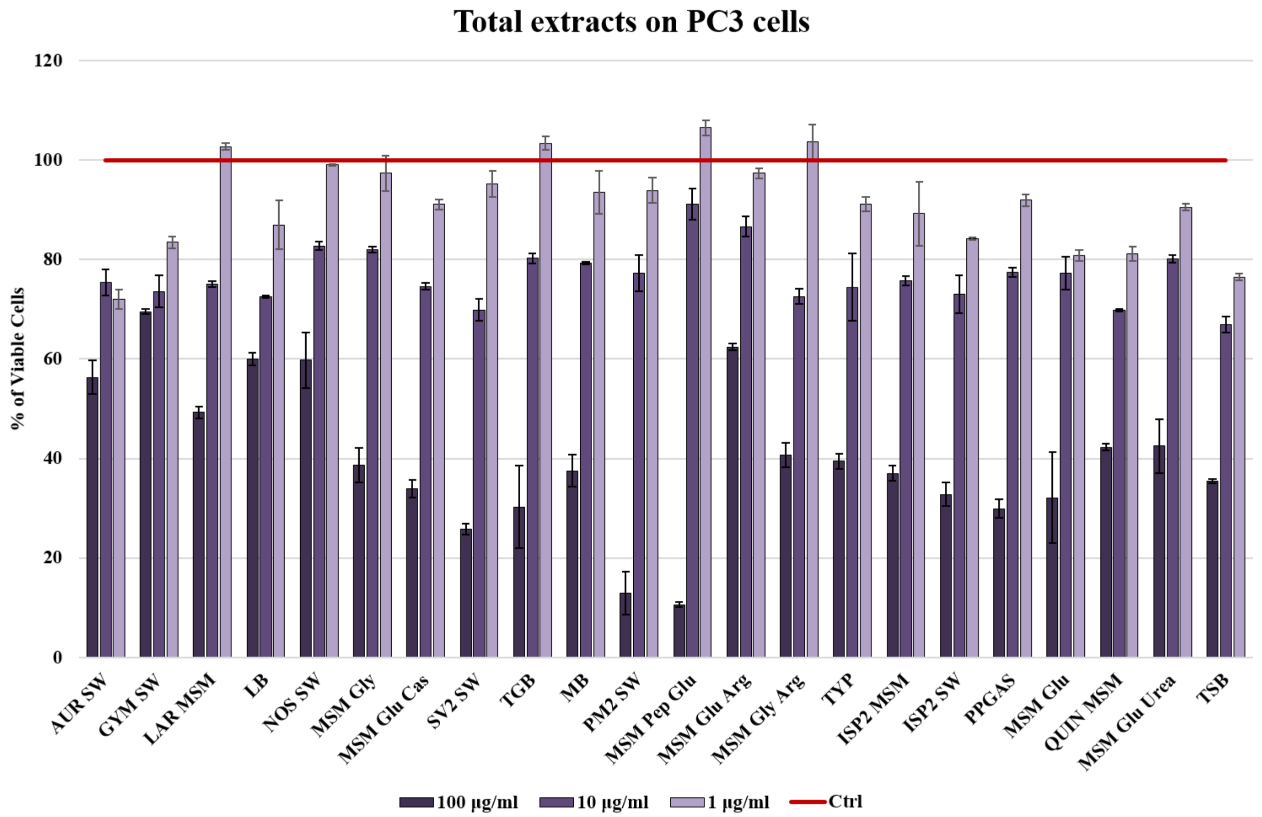

Figure S1. Antiproliferative assay of crude extracts towards PC3 cells (cancer prostatic cell line). Cells were treated with 1, 10 and 100 µg/mL of total ex-tracts for 48 hours. Untreated cells were used as control and corresponded to 100% of cell viability (red horizontal line).

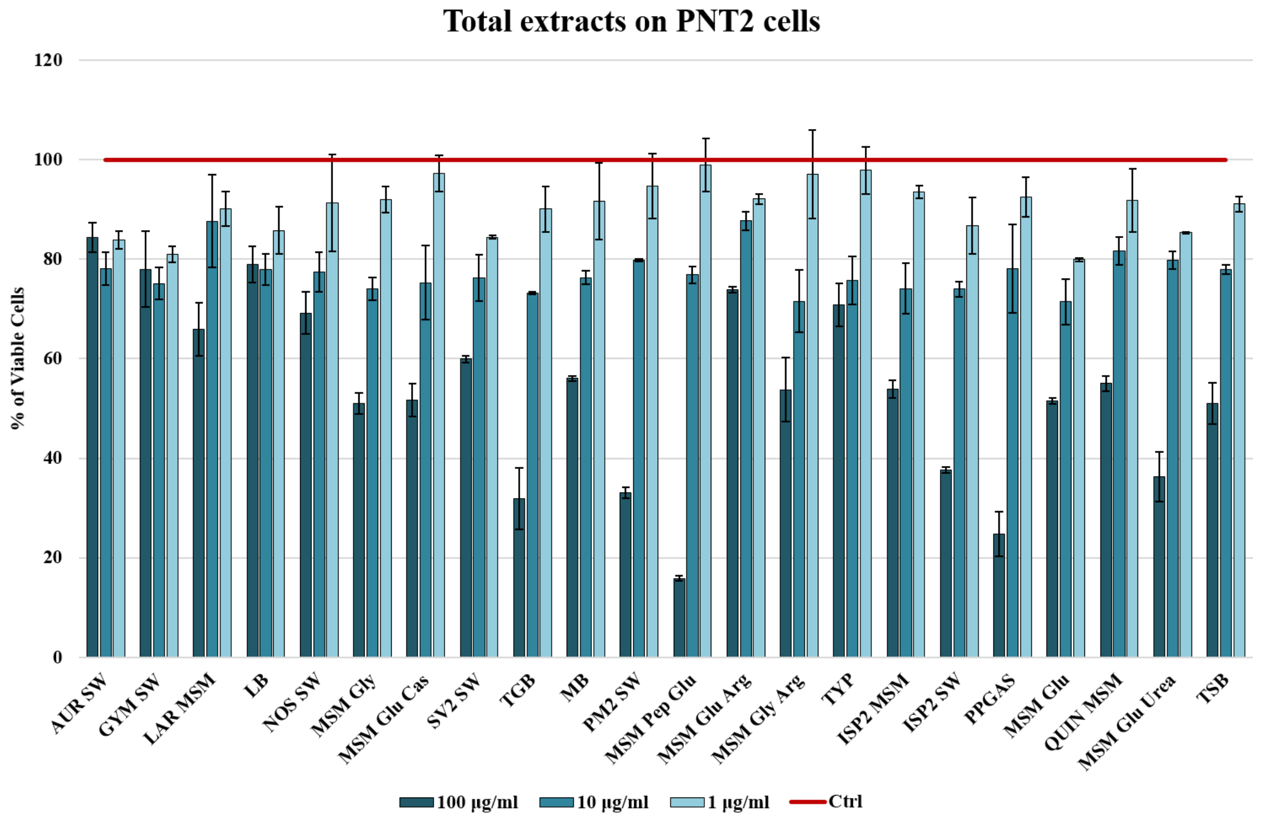

Figure S2. Viability assay on PNT2 (human normal prostatic cell line). Cells were treated with 1, 10 and 100 µg/mL of total ex-tracts for 48 hours. Untreated cells were used as control and corresponded to 100% of cell viability (red horizontal line).

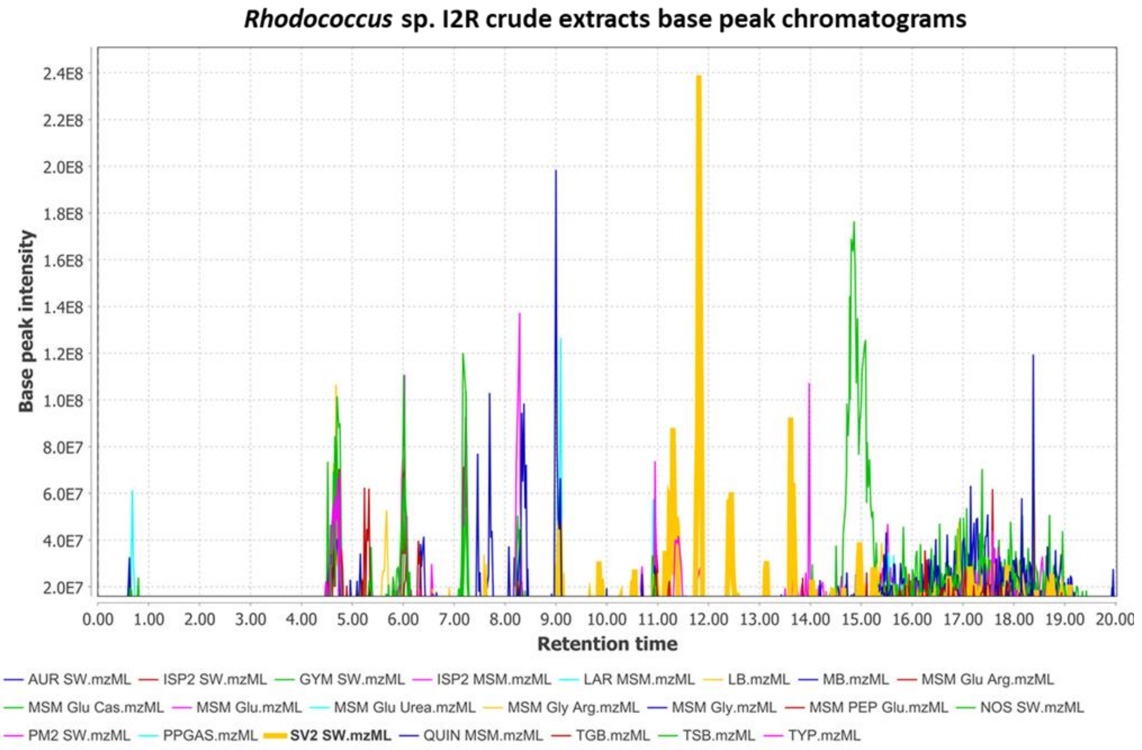

Figure S3. *Rhodococcus* sp. I2R crude extracts base peak chromatograms. Each colour indicates a crude extract analysed by LC-MS and processed through MZmine. Bold orange indicates the extract SV2 SW.

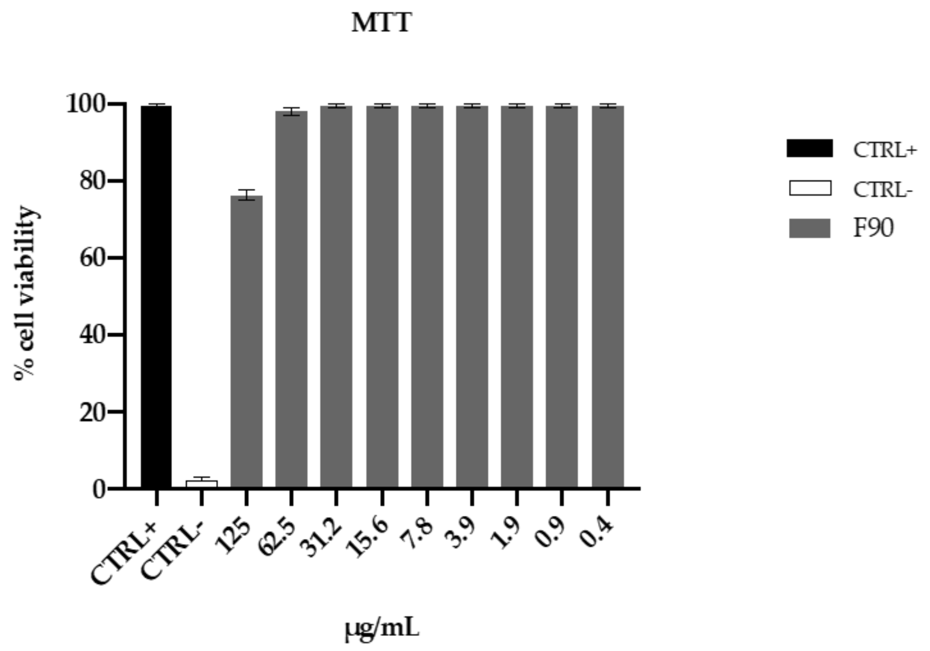

Figure S4. Viability assay on Vero cells. Cells were treated with decreasing concentration of 90% MeOH fraction, from 125 to 0.4 µg/mL. Greco extract was used as positive control (CTRL+). Untreated cells represent the negative control (CTRL-).

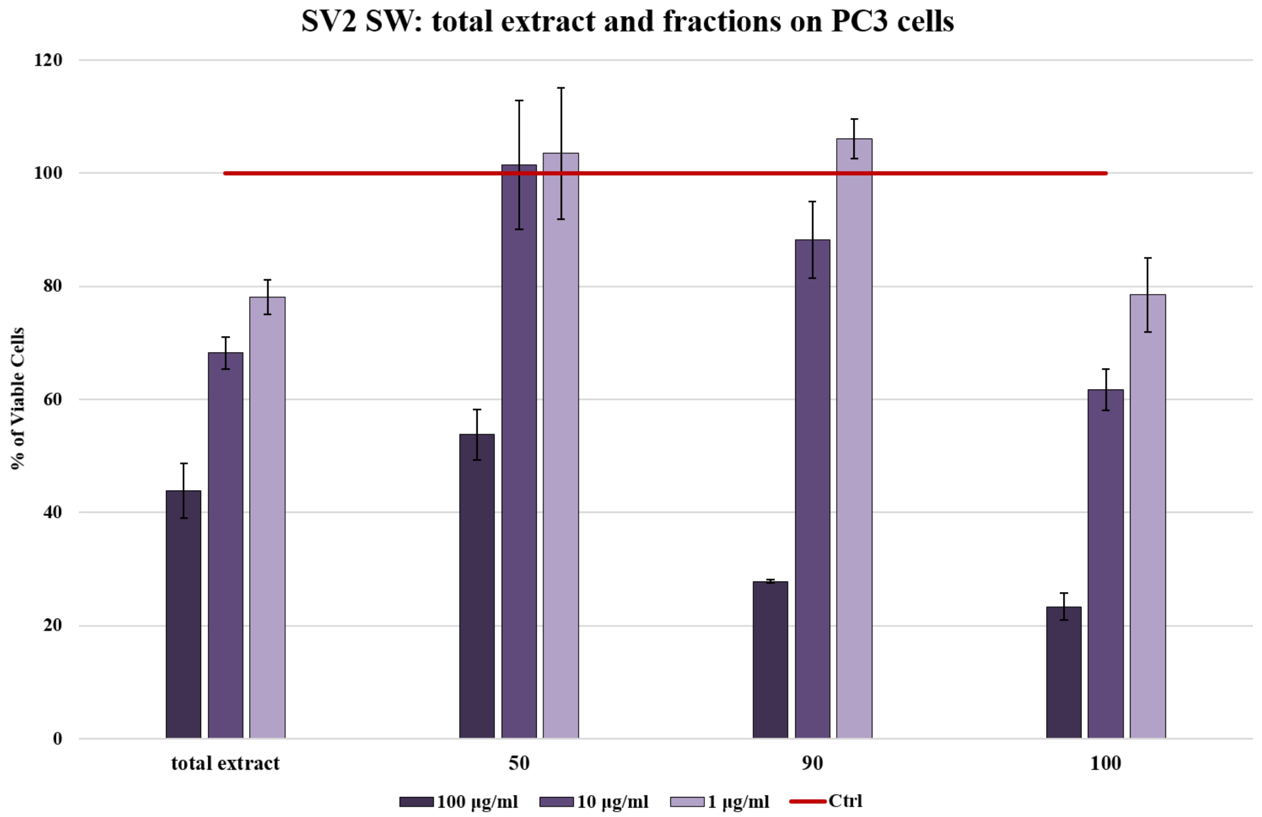

Figure S5. Antiproliferative assay of SV SW fractions towards PC3 cells. F50, F90, and F100 were tested at three different concentrations (100 µg/mL, 10 µg/mL, 1 µg/mL). Untreated cells were used as control and corresponded to 100% of cell viability (red horizontal line).

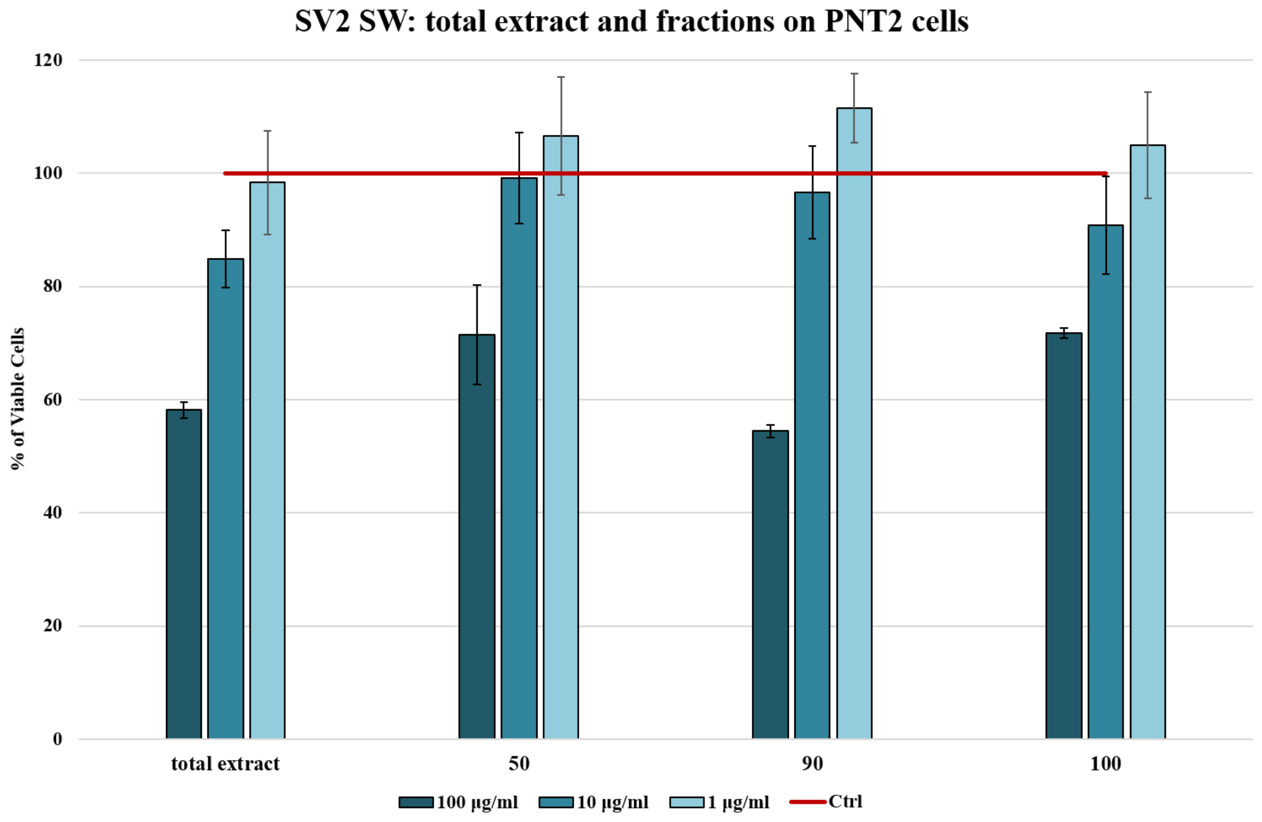

Figure S6. Viability assay of SV2 SW fractions towards PNT2 cells. F50, F90, and F100 were tested at three different concentrations (100 µg/mL, 10 µg/mL, 1 µg/mL). Untreated cells were used as control and corresponded to 100% of cell viability (red horizontal line).

Table S1. Succinyl saccharide esters from *Rhodococcus* sp. I2R.

	R_t (min.)	$[M+H]^+$	m/z	Primary acyl chains		Secondary acyl chains [a]
**disaccharide succinic diesters**	14.6	$C_{26}H_{45}O_{16}$	613.2696	3-OH-C10		-
	15.4	$C_{25}H_{43}O_{15}$	583.2591	C9		-
	16.0	$C_{27}H_{47}O_{16}$	627.2853	3-OH-C11		-
	16.3	$C_{26}H_{43}O_{15}$	595.2591	C10:1		-
	16.7	$C_{26}H_{45}O_{15}$	597.2744	C10		-
	18.0	$C_{32}H_{47}O_{17}$	703.2798	3-OH-C8 [b]		PhAc
	19.3	$C_{30}H_{53}O_{16}$	669.3320	3-OH-C14		-
	19.9	$C_{34}H_{51}O_{17}$	731.3108	3-OH-C10		PhAc
**disaccharide succinic triesters**	16.6	$C_{34}H_{51}O_{18}$	747.3066	3-OH-C8	3-OH-PhBu	-
	16.9	$C_{32}H_{47}O_{17}$	703.2798	3-OH-C8	PhAc	-
	17.5	$C_{32}H_{55}O_{18}$	727.3378	3-OH-C8	3-OH-C8	-
	17.8	$C_{40}H_{61}O_{20}$	861.3744	3-OH-C8	diOH-C8	PhAc
	18.5	$C_{33}H_{57}O_{18}$	741.3530	3-OH-C9	3-OH-C8	-
	19.0	$C_{34}H_{51}O_{17}$	731.3116	3-OH-C10	PhAc	-
	19.4	$C_{34}H_{59}O_{18}$	755.3685	3-OH-C10	3-OH-C8	-
	19.8	$C_{38}H_{57}O_{19}$	817.3481	3-OH-C8	3-OH-C6	PhAc [c]
	20.8	$C_{39}H_{59}O_{19}$	831.3633	3-OH-C8	3-OH-C7	PhAc [c]
	20.9	$C_{42}H_{57}O_{19}$	865.3478	3-OH-C8	3-OH-PhBu	PhAc

	21.6	$C_{40}H_{61}O_{19}$	845.3790	3-OH-C8	3-OH-C8		PhAc	
	22.4	$C_{41}H_{63}O_{19}$	859.3952	3-OH-C9	3-OH-C8		PhAc [c]	
	25.2	$C_{44}H_{69}O_{19}$	901.4421	3-OH-C10	3-OH-C10		PhAc	
**trisaccharide succinic diesters**	17.2	$C_{38}H_{57}O_{22}$	865.3325	3-OH-C8			PhAc	
**trisaccharide succinic triesters**	18.0	$C_{40}H_{69}O_{23}$	917.4213	3-OH-C10	3-OH-C8		-	
	18.4	$C_{48}H_{75}O_{25}$	1051.4582	3-OH-C10	diOH-C8		PhAc	
	19.5	$C_{48}H_{67}O_{24}$	1027.4005	3-OH-C8	3-OH-PhBu		PhAc	
	20.1	$C_{44}H_{75}O_{24}$	987.4641	3-OH-C8	3-OH-C8		C6	
	20.1	$C_{46}H_{71}O_{24}$	1007.4326	3-OH-C8	3-OH-C8		PhAc	
	20.8	$C_{47}H_{73}O_{24}$	1021.4476	3-OH-C9	3-OH-C8		PhAc [c]	
	21.5	$C_{48}H_{75}O_{24}$	1035.4631	3-OH-C10	3-OH-C8		PhAc [c]	
	22.2	$C_{49}H_{77}O_{24}$	1049.4792	3-OH-C10	3-OH-C9		PhAc	
	22.2	$C_{49}H_{77}O_{24}$	1049.4792	3-OH-C11	3-OH-C8		PhAc	
	23.1	$C_{50}H_{79}O_{24}$	1063.4945	3-OH-C10	3-OH-C10		PhAc	
	23.6	$C_{55}H_{79}O_{25}$	1139.4904	3-OH-C9	3-OH-C8		PhAc	PhAc [d]
	23.7	$C_{52}H_{81}O_{25}$	1105.5056	3-OH-C8	3-OH-C8		PhAc	C6 [d]
	24.6	$C_{53}H_{83}O_{25}$	1119.5217	3-OH-C9	3-OH-C8		PhAc	C6 [d]
	26.3	$C_{55}H_{87}O_{25}$	1147.5524	3-OH-C11	3-OH-C8		PhAc	C6 [d]
	26.3	$C_{55}H_{87}O_{25}$	1147.5524	3-OH-C10	3-OH-C9		PhAc	C6 [d]
**trisaccharide succinic tetraesters**	27.9	$C_{54}H_{85}O_{24}$	1117.5419	3-OH-C8	C10	C6 [e]	PhAc	
	29.4	$C_{58}H_{91}O_{26}$	1203.5785	3-OH-C8	3-OH-C8	C6 [e]	PhAc	C6 [d]

Abbreviations: *PhAc*, phenyl acetate; *3-OH-PhBu*, 3-OH-4-phenylbutanoate.

[a] secondary acyl chains linked to 3-OH-fatty acids as indicated in Figure 9.

[b] 3-OH FAs with OH highlighted in red bear a secondary acyl chain through *O*-ester linkage.

[c] mixture of isomers differing for the position of the secondary acyl chain which can be linked either to 1st or 2nd primary acyl chain.

[d] compounds displaying two secondary acyl chains on the 1st and 2nd primary acyl chains, respectively.

[e] compounds featuring a third sugar unit which bears a hexanoate unit (C6).

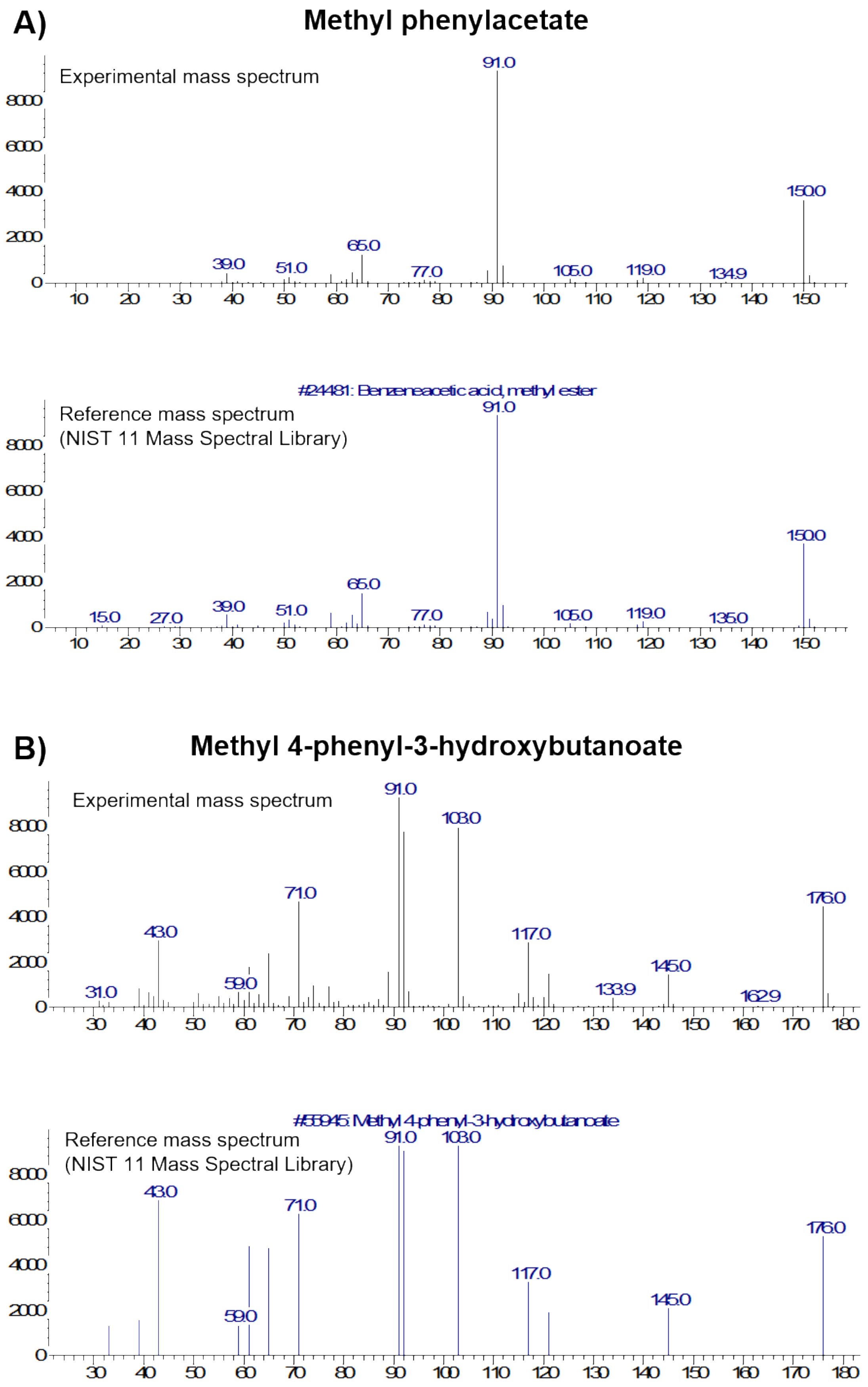

Figure S7. GC/MS mass spectra of methyl phenylacetate (**A**) and methyl 4-phenyl-3-hydroxybutanoate (**B**) obtained after methanolysis of the 90% MeOH fraction from crude extract SV2 SW.

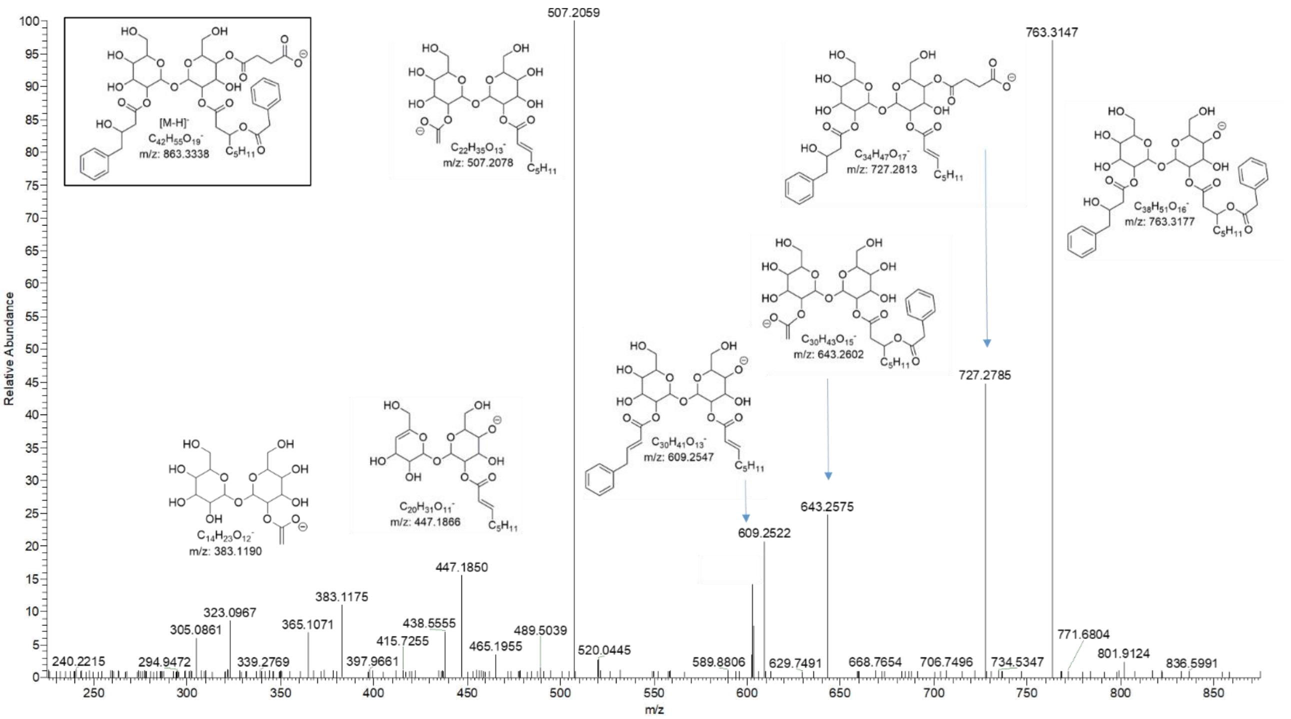

Figure S8. HR-MS/MS spectrum of a representative succinic disaccharide triester (*m/z* 863.3309, $C_{42}H_{55}O_{19}^-$) from *Rhodococcus* sp. I2R, bearing an unusual 3-OH-4-phenylbutanoate moiety.

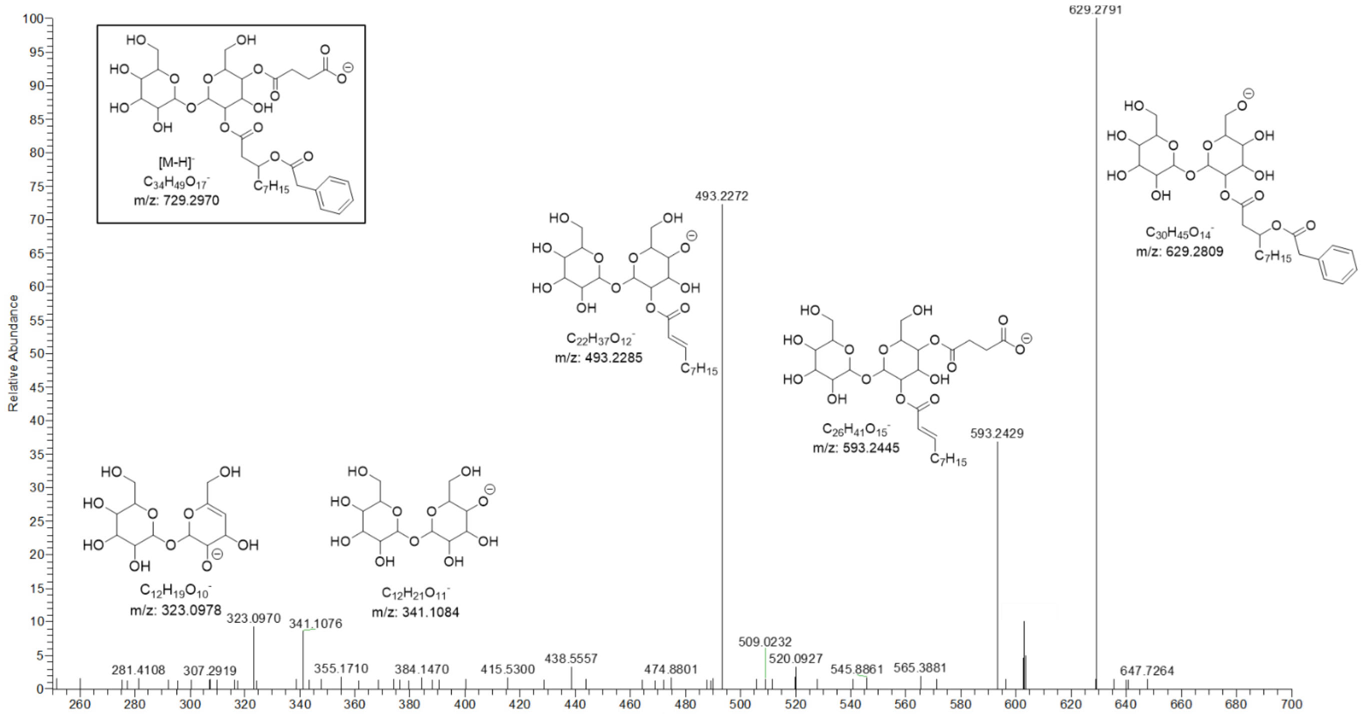

Figure S9. HR-MS/MS spectrum of a representative succinic disaccharide diester (*m/z* 729.2948, $C_{34}H_{49}O_{17}^-$) from *Rhodococcus* sp. I2R.

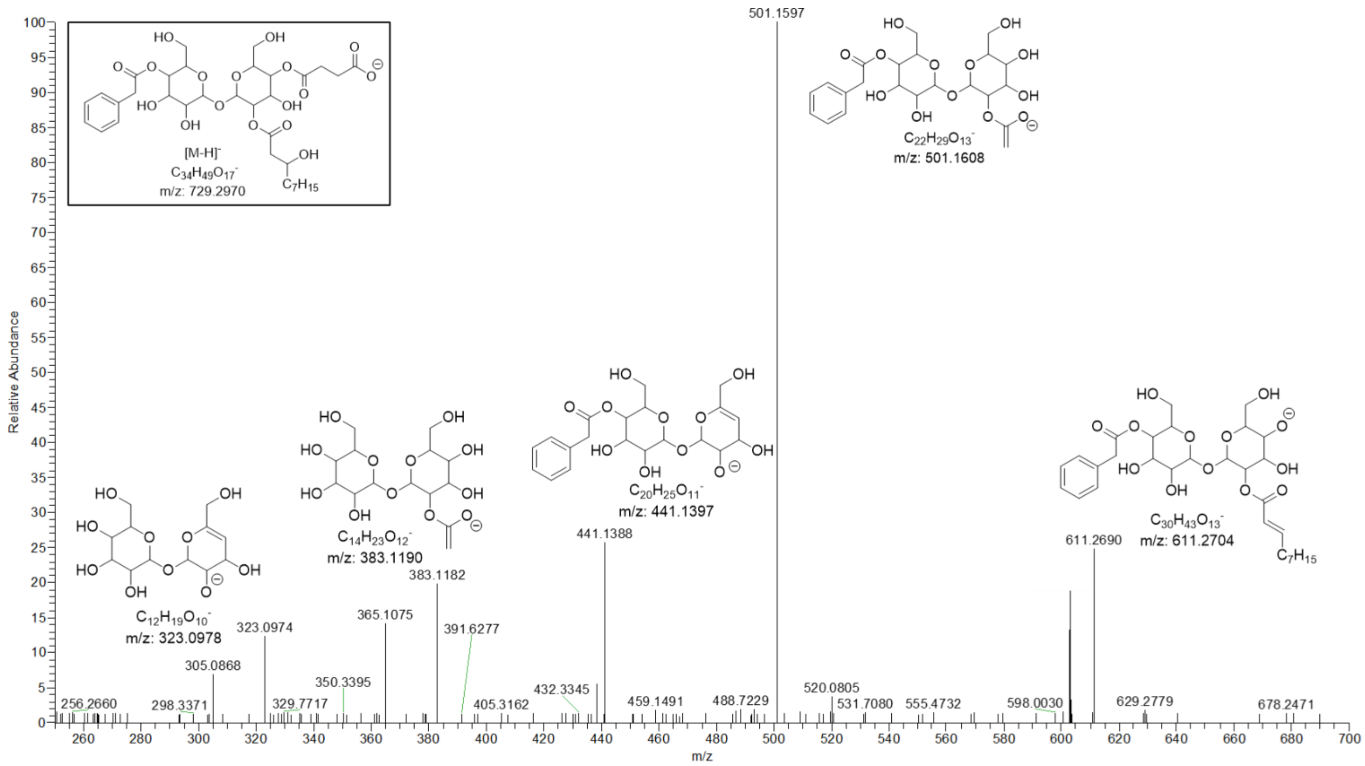

Figure S10. HR-MS/MS spectrum of a representative succinic disaccharide triester (*m/z* 729.2944, $C_{34}H_{49}O_{17}^-$) from *Rhodococcus* sp. I2R.

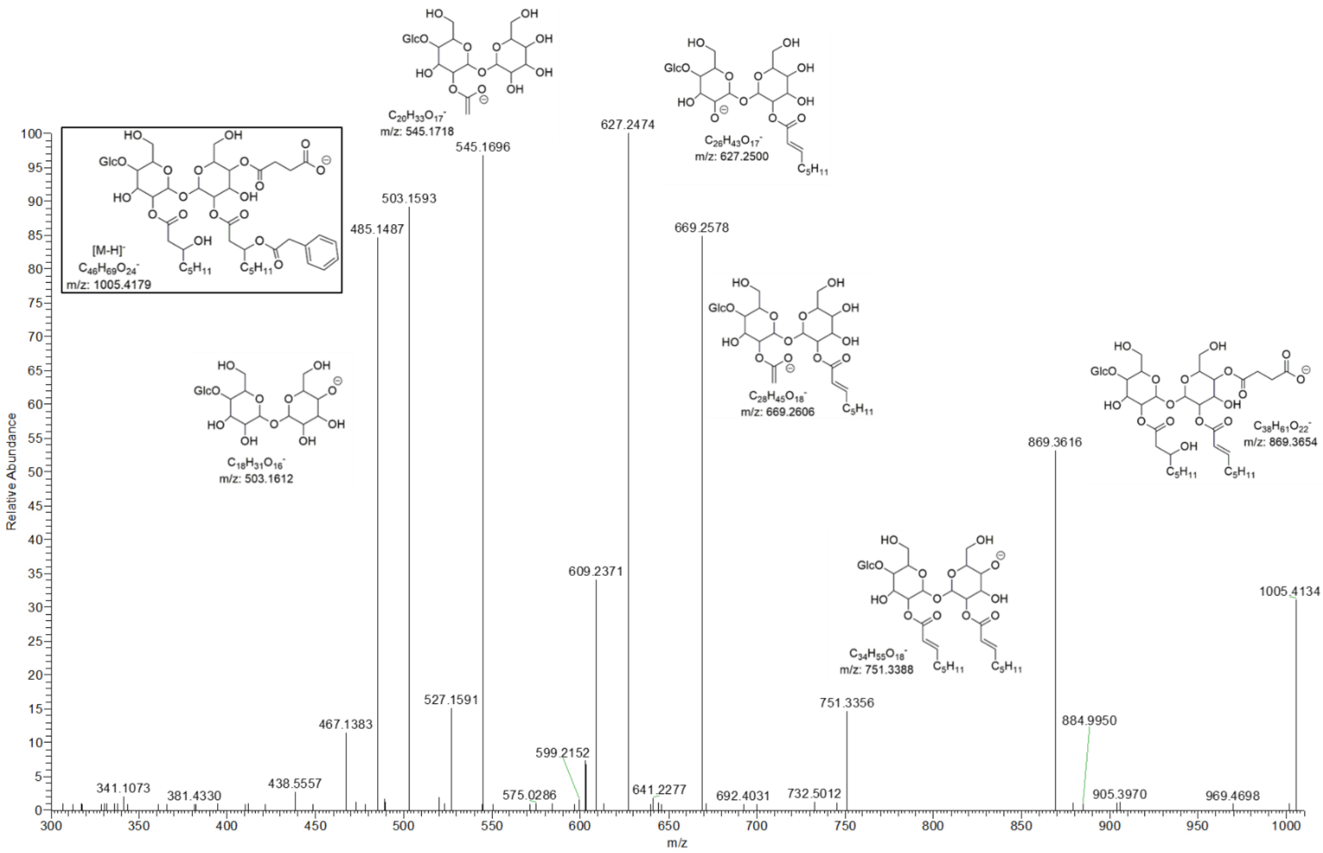

Figure S11. HR-MS/MS spectrum of a representative succinic trisaccharide triester (*m/z* 1005.4134, $C_{46}H_{69}O_{24}^-$) from *Rhodococcus* sp. I2R.

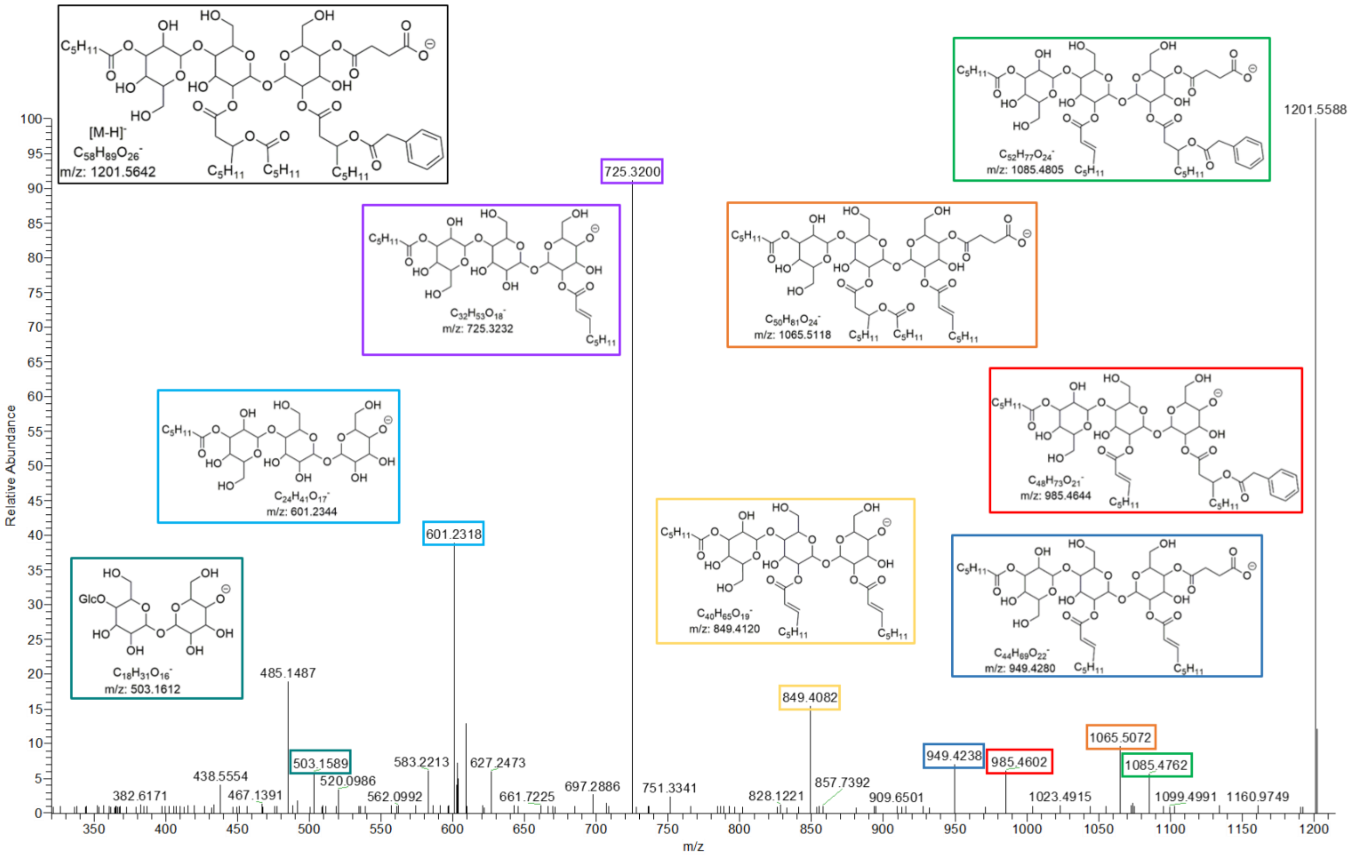

Figure S12. HR-MS/MS spectrum of a representative succinic trisaccharide tetraester (*m/z* 1201.5588, $C_{58}H_{89}O_{26}^-$) from *Rhodococcus* sp. I2R.
